# Prognostic value of miR-21 for prostate cancer: a systematic review and meta-analysis

**DOI:** 10.1042/BSR20211972

**Published:** 2022-01-11

**Authors:** M.Y. Cynthia Stafford, Colin E. Willoughby, Colum P. Walsh, Declan J. McKenna

**Affiliations:** 1 Genomic Medicine Research Group, Ulster University, Cromore Road, Coleraine, BT52 1SA, U.K.; 2 Centre for Research and Development, Region Gävleborg/Uppsala University, Gävle, Sweden

**Keywords:** meta analysis, microRNA, miR-21, prognostic, prostate cancer, systematic

## Abstract

Elevated levels of miR-21 expression are associated with many cancers, suggesting it may be a promising clinical biomarker. In prostate cancer (PCa), however, there is still no consensus about the usefulness of miR-21 as an indicator of disease progression. This systematic review and meta-analysis was conducted to investigate the value of miR-21 expression as a prognostic measurement in PCa patients. Medline (Ovid), EMBASE, Web of Science, Scopus and Cochrane Library databases were systematically searched for relevant publications between 2010 to 2021. Studies exploring the relationship between miR-21 expression, PCa prognosis and clinicopathological factors were selected for review. Those reporting hazard ratio (HR) and 95% confidence intervals (CIs) were subject to meta-analyses. Fixed-effect models were employed to calculated pooled HRs and 95% CIs. Risk of bias in each study was assessed using QUIPS tool. Certainty of evidence in each meta-analysis was assessed using GRADE guidelines. A total of 64 studies were included in the systematic review. Of these, 11 were eligible for inclusion in meta-analysis. Meta-analyses revealed that high miR-21 expression was associated with poor prognosis: HR = 1.58 (95% CI = 1.19–2.09) for biochemical recurrence, MODERATE certainty; HR = 1.46 (95% CI = 1.06–2.01) for death, VERY LOW certainty; and HR = 1.26 (95% CI = 0.70–2.27) for disease progression, VERY LOW certainty. Qualitative summary revealed elevated miR-21 expression was significantly positively associated with PCa stage, Gleason score and risk groups. This systematic review and meta-analysis suggests that elevated levels of miR-21 are associated with poor prognosis in PCa patients. miR-21 expression may therefore be a useful prognostic biomarker in this disease.

## Introduction

Prostate cancer (PCa) is the most commonly diagnosed cancer for males in 105 countries including North and South America, Western Europe and Australia [1]. The majority of PCa cases are localized disease with very high survival rate after initial treatment (∼100% 5-year survival), but recurrence may occur in about 40% as biochemical recurrence (BCR) or distant metastasis that has a significantly poorer prognosis (∼30% 5-year survival) [2]. Additionally, some may progress as castration-resistant prostate cancer (CRPC) or develop chemoresistance [3].

Currently, prognosis is predicted by considering cancer stage, Gleason score, prostate-specific antigen (PSA) level, patient’s health condition, treatment choice and treatment response [4]. However, these clinicopathological factors still have certain limitations. For example, Gleason score is a histological method which is subject to inter-observer variability, and clinicians can find the grading system confusing [5,6]. Staging may vary between clinical and pathological estimation, forcing clinicians to alter treatment regime, and prognosis for lower stage cancer is less than predictable [7]. PSA lacks specificity and BCR, defined by rise in PSA level following prostatectomy or radiotherapy, does not necessarily predict clinical recurrence or metastasis with sufficient accuracy [8]. Therefore, there is still a clear clinical need for novel molecular markers that may overcome some of these limitations [9].

MicroRNAs (miRNAs) are a class of non-coding molecules which have emerged as strong candidates for useful clinical biomarkers [10]. Over the past decade, they have been actively researched in a wide range of diseases, including prostate cancer [11,12]. miRNAs are estimated to regulate 60% of gene expression in human and some specifically target oncogenes or tumor suppressor genes [10,11]. The aberrant expression of miRNAs can therefore contribute to cancer development and several dysregulated miRNAs have been associated with PCa progression [12,13]. Importantly, miRNAs can be detected in blood and urine, as well as tissue. Indeed, they are known to be more stable in biofluids than other nucleic acids which give them potential as diagnostic or prognostic markers [13,14]. However, more research is needed to understand which miRNAs are most relevant in prostate cancer.

miR-21 is one of the most studied miRNAs and there is a large body of evidence to suggest that it has a predominantly oncogenic function since it is over-expressed in many cancers [14]. As one of the first miRNAs to be categorized as an ‘oncomiR’, it has been subsequently evaluated for its potential use as a clinical biomarker in various cancers [15–17]. Several recent systematic reviews have found evidence that circulating miR-21 levels can predict poor prognosis in esophageal, pancreatic, colorectal and breast cancers [18,19]. In urological cancers, including PCa, Chen et al. found some evidence that miR-21 over-expression was significantly associated with unfavorable prognosis in their integrated analysis [20]. However, despite evidence that it can contribute to PCa development, no systematic review or meta-analysis to date has been carried out specifically for miR-21 in this setting. Therefore, the purpose of this paper is to systematically evaluate studies related to prognostic value of miR-21 in PCa, appraising study qualities and synthesising evidence by meta-analyses, data association and qualitative summary.

## Materials and methods

### Protocol and registration

This review was conducted following a protocol which was registered with the International Prospective Register of Systematic Reviews (PROSPERO; https://www.crd.york.ac.uk/prospero/) under the registration ID: CRD42020183408 on 23 June 2020. The protocol was developed following guidance on PRISMA-P [21], systematic review and meta-analysis of prognostic factor studies [22] and the checklist of items recommended in the PRISMA statement [23].

### Search strategy

Electronic databases from which records were retrieved include Medline (Ovid), EMBASE, Web of Science, Scopus and Cochrane Library, covering publications from 2010 to 2021 and they were last searched on 8 November 2021. Additionally, reference lists of included studies and relevant review papers were searched manually. Prognostic factor studies were prone to selective reporting in that miRNAs with insignificant findings might not be reported [24]; therefore, a high-sensitivity approach was used in the search strategy as shown in Supplementary Table ST 1. Key words related to miRNAs, in addition to miR-21, were included to broaden the search to cover relevant studies that measured miR-21 but did not report the result. Retrieved records from databases were exported to systematic review manager *Rayyan* where duplicates were removed [25]. Titles and abstracts of remaining records were screened for relevance independently by two reviewers. Full text of studies selected for inclusion were subsequently imported into another systematic review manager *Covidence* (www.covidence.org) where studies were assessed for eligibility in duplicate. Any disagreements were resolved through discussion.

### Eligibility criteria

For inclusion in the systematic review, original peer-reviewed human studies published in English from year 2010 to 2021 with full-text available online or from Ulster University Library were included. *In vitro*, *in silico* and *in vivo* studies that did not include human participants were excluded. Studies without original human data that analyzed publicly available human data (e.g., from The Cancer Genome Atlas repository) were not included to avoid multiple counting of sample size. Review-type studies and duplicate reports were excluded for the same reason. If the same study was published in multiple journals, only the most informative or the most recent one was included. Studies published before 2010 were excluded due to advances in miRNA technology.

For meta-analyses, studies with characteristics specified by PICOT (Table 1) were eligible for inclusion in meta-analysis [22]. Length of follow-up was not restricted to broaden the number of inclusions and increase the number of eligible studies.

**Table 1:** PICOT eligibility criteria

P	Population	Male patients of any age worldwide diagnosed with PCa.
**I**	Index prognostic factor	Measurement of miR-21 levels in tissue or circulating/fluid samples such as tumour tissue, blood, plasma, serum, urine and seminal fluid.
**C**	Comparator prognostic factors	Clinicopathological factors such as stage, grade, Gleason score, PSA level and health condition (e.g., recurrence, metastasis).
**O**	Outcomes of interest	Survival outcomes of any type (e.g., OS, RFS) estimated in HR, 95% CI, *P*-value and/or survival curves with log-rank *P*-value.
**T**	Timing	Samples taken as baseline at the start of follow-up of any length.

Studies with characteristics specified by PICOT were eligible for inclusion in meta-analysis.

Abbreviations: CI, confidence interval; HR, hazard ratio; OS, overall survival; PCa, prostate cancer; PSA, prostate-specific antigen; RFS, recurrence-free survival.

### Data collection process

A data extraction form adapted from CHARMS-PF checklist [22] was created within *Covidence* to capture information about each study, source of data, PICOT details, sample size, missing data, statistical analysis methods, survival outcome results and/or association analysis results (Supplementary Table ST 2). Data were extracted independently in duplicate into separate forms. Completed forms were compared, and conflicts were resolved through discussion. Authors of 12 studies were contacted for missing data or clarifications (Supplementary Table ST 3). Only data relevant to prognosis were considered; therefore, data related to diagnosis and healthy or benign prostatic hyperplasia (BPH) controls were disregarded.

### Risk of bias in individual studies

Judgment was made independently in duplicate using the Quality in Prognostic Factor Studies (QUIPS) tool that assesses risk of bias as HIGH, MODERATE, LOW or UNCLEAR in six domains (Supplementary Table ST 4) [26]. For domain 3 ‘Prognostic factor measurement’, methods accepted as reliable for miR-21 measurement were qPCR, sequencing and array technology. For domain 5 ‘Adjustment for covariates’, the core set of desired adjustment covariates was predefined as Gleason score/grade and pathological/clinical stage.

### Statistical analysis

The principal summary measure for meta-analysis was hazard ratio (HR), presented with 95% confidence interval (CI) and *P*-value. Kaplan–Meier plot presented with log-rank *P*-value was also accepted. Eligible studies of similar design in terms of outcome and handling of miR-21 data were grouped into separate meta-analyses. For each meta-analysis effect estimates were pooled as HR (95% CI) based on fixed-effect inverse variance method in the review manager *RevMan5.4* [27]. Statistical heterogeneity was assessed by visual inspection of the forest plot, chi-square (Chi^2^) test and *I*^2^ test (Chi^2^ *P*≤0.1 indicates significant heterogeneity; *I*^2^<30% denotes low/unimportant heterogeneity, 30–60% moderate heterogeneity, 50–90% substantial heterogeneity and 75–100% considerable heterogeneity). Impact on the robustness of analyses by the presence of an outlier and the inclusion of a study that introduced clinical heterogeneity was assessed by sensitivity analyses. For qualitative summary, association measure included but was not limited to correlation, fold change (FC) or mean difference.

### Certainty of evidence

For each analysis the certainty of evidence was rated according to the Grading of Recommendations Assessment, Development and Evaluation (GRADE) guidelines [28]. This review estimated the prognostic value of miR-21 in PCa as an exploratory study without direct association with clinical decision making; therefore, certainty was rated based on the non-contextualized setting as HIGH, MODERATE, LOW or VERY LOW certainty. Starting from HIGH certainty, evidence could be rated down in five domains: risk of bias, inconsistency, indirectness, imprecision and publication bias; or rated up in three domains: large effect, dose−response and plausible confounding. Assessment of publication bias was not possible due to low number of studies eligible for each analysis, which meant any test of bias would be underpowered.

## Results

### Study selection

**Figure 1:**
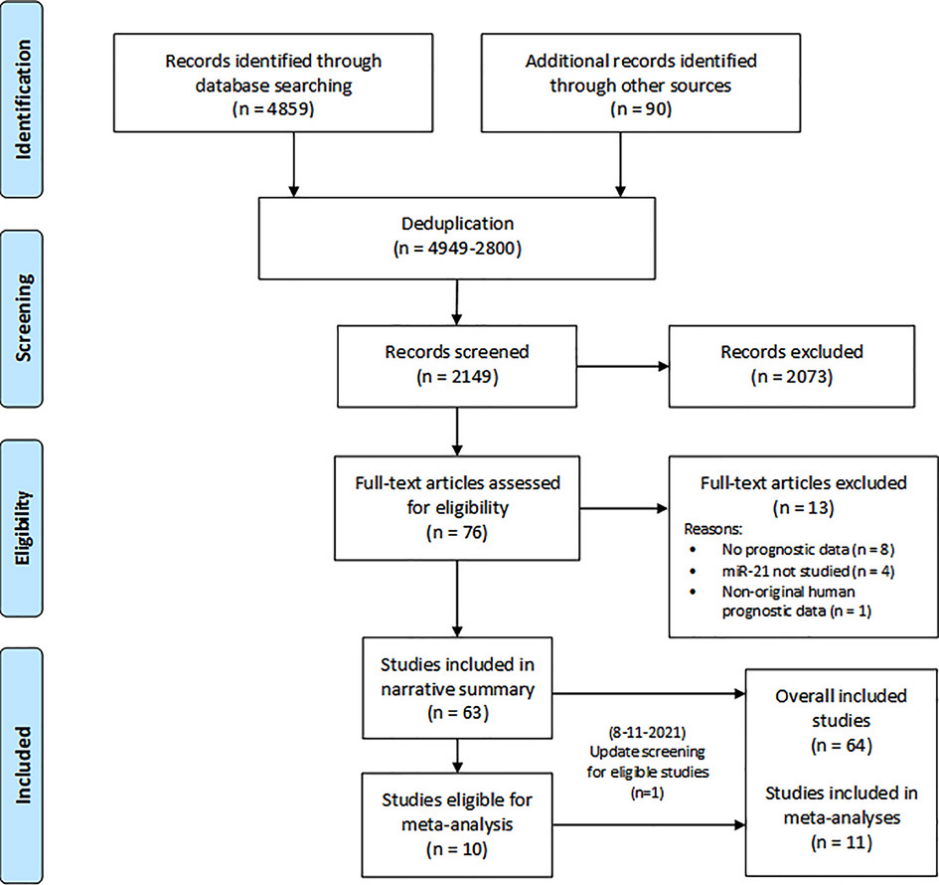
Study flow diagram (adapted from Moher et al. [23])

Study selection was as shown in the flow diagram (Figure 1). Up until 23 July 2020, 4859 records were retrieved from database searching and a further 90 were identified from manual searching of reference lists of included studies and relevant reviews. After duplicates were removed (*n*=2800), record screening identified 76 eligible studies for full-text assessment. Thirteen full-text articles were ineligible due to lack of prognostic data (*n*=8), lack of miR-21 data (*n*=4) and lack of original human prognostic data (*n*=1) (Supplementary Table ST 5). The remaining 63 studies [29–77,79–92] were included in the systematic review, with 10 eligible for meta-analysis. On 8 November 2021, an update screening for meta-analysis identified one more eligible study [78], bringing the total number of included studies to 64, with 11 eligible for meta-analysis.

### Study characteristics

Characteristics of all 64 studies included in this systematic review are summarized in Supplementary Table ST 6. Each included study was assigned a Study ID composed of first author’s name and publication year. The PICOT eligibility criteria (Table 1) identified studies on PCa patient cohorts which could be stratified against measurable parameters and outcomes for inclusion in the meta-analysis. A total of 11 studies, with study sizes ranging from 31 to 478 participants, encompassing 1485 PCa patients total, were eligible for meta-analysis (Tables 2 and 3). Amankwah, 2013 [31] indicated that the recurrent group was oversampled, no rationale was provided. Sharova, 2021 [78] was clearly indicated as prospective; Zedan, 2017 [85] and Zhao, 2019a [89] were clearly indicated as retrospective studies. Cohort types were projected for the rest judging by the details contained. Thus, six studies appeared to be prospective (Guan, 2016 [42]; Leite, 2015 [60]; Lin, 2014 [64]; Lin, 2017 [65]; Sharova, 2021 [78]; Yang, 2016 [84]) and four were retrospective (Amankwah, 2013 [31]; Melbø-Jørgensen, 2014 [68]; Zedan, 2017 [85]; Zhao, 2019a [89]); it was unclear for Li, 2012 [61].

**Table 2:** Characteristics of 11 studies eligible for meta-analyses

Study ID, Type	Study size	P	I	C	O	Follow-up period
Amankwah, 2013 [31] *Retrospective*	65	PCa histologically confirmed; Underwent RP Non-recurrent median age = 59 (47–75) Recurrent (oversampled) median age = 57 (46–75)	High/low miR-21, -221 & -222 in FFPE tissue (TaqMan RT-qPCR)	Age; BMI; cT; GS	RFS^4^	3–254 months
Guan, 2016 [42] *Prospective*	85	PCa pathologically confirmed; Underwent ADT^1^ Mean age = 75 ± 7.7	High/low levels of 7 miRNAs (including miR-21) in FFPE tissue (TaqMan RT-qPCR)	Age; cT; GS; PSA	PFS^5^	14–95 months
Leite, 2015 [60] *Prospective*	127	Localized PCa; Underwent RP Mean age = 63 ± 7.6	High/low miR-21 in FFPE tissue (TaqMan RT-qPCR)	GG; PSA; pT	RFS^6^	2–120 months
Li, 2012 [61] *(unclear)*	168	PCa pathologically confirmed; Underwent RP and regional lymph node dissection Low miR-21 median age = 68 (56–77) High miR-21 median age = 67 (48–77)	High/low miR-21 in FFPE tissue (LNA-ISH)	Age; Capsular invasion; GS; pN; PSA; pT; Surgical margin	RFS^6^	2–80 months
Lin, 2014 [64] *Prospective*	97	CRPC patients; Underwent docetaxel chemotherapy Median age = 68 (46–87)	High/low levels of 46 miRNAs (including miR-21) in plasma/serum (TaqMan RT-qPCR)	Age; Alkaline phosphatase; GS; Haemoglobin; PSA; Visceral metastasis	OS	3–62 months
Lin, 2017 [65] *Prospective*	87	CRPC patients; Underwent docetaxel chemotherapy Median age = 72 (40–89)	High/low levels of 14 miRNAs (including miR-21) in plasma (TaqMan RT-qPCR)	Alkaline phosphatase; Hemoglobin; PSA	OS	0.7–45 months
Melbø-Jørgensen, 2014 [68] *Retrospective*	478	PCa patients; Underwent RP Median age = 62 (45–75)	High/low levels of 7 miRNAs (including miR-21-5p) in FFPE tissue (RT-qPCR)	GG; Perineural infiltration; PSA; pT; Surgical margins; Tumor size; Vascular infiltration	RFS^7^	6–188 months
Sharova, 2021 [78] *Prospective*	31	mCRPC patients; Treated with ARTA^2^ Median age = 75 (69.5–80.5)	High/low levels of miR-21, -141 & -223 in plasma (TaqMan RT-qPCR)	GS; Hemoglobin; Neutrophil/lymphocyte ratio; PSA; Time to CRPC	OS PFS^8^	Median = 36.6 months
Yang, 2016 [84] *Prospective*	92	PCa pathologically confirmed; Underwent resection Mean age = 60 ± 6	High/low miR-21 in PBMC (TaqMan RT-qPCR)	Age; cT; GS; PSA	OS	21–69 months
Zedan, 2017 [85] *Retrospective*	49	Localised PCa; Underwent RP & regional lymph node dissection Mean age = 62.7 (52–71)	Continuous levels of 6 miRNAs (including miR-21) in FFPE tissue (ISH analysed by computer software)	GS; PSA; pT	RFS^6^	(Not stated)
Zhao, 2019a [89] *Retrospective*	206	PCa patients; Underwent RP Median age = 63 (47–74)	Continuous levels of 20 miRNAs (including miR-21-5p) in FFPE tissue (TaqMan RT-qPCR)	Age; DRE; PSA; ISUP grade^3^; pN; Prostate volume; pT; Surgical margin	RFS^6^	17–180 months

Abbreviations: ADT, androgen deprivation therapy; ARTA, androgen receptor-targeted agents; BMI, body mass index; C, comparator prognostic factors; CRPC, castration-resistant prostate cancer; cT, clinical tumor stage; DRE, digital rectal examination; FFPE, formalin-fixed paraffin-embedded; GG, Gleason grade; GS, Gleason score; I, index prognostic factor; ISH, *in situ* hybridization; ISUP, International Society of Urological Pathology; LNA-ISH, locked nucleic acid *in situ* hybridization; mCRPC, metastatic castration-resistant prostate cancer; O, outcomes of interest; OS, overall survival; P, population; PBMC, peripheral blood mononuclear cell; PCa, prostate cancer; PFS, progression-free survival; pN, lymph node metastasis; PSA, prostate-specific antigen; pT, pathological tumor stage; RFS, recurrence-free survival; RP, radical prostatectomy; RT-qPCR, real-time quantitative polymerase chain reaction.

^1^ADT included surgical castration or luteinising hormone-releasing hormone agonist combined with an antiandrogen according to Guan, 2016 [42].

^2^ARTA included abiraterone (*n*=10) and enzalutamide (*n*=21) according to Sharova, 2021 [78].

^3^ISUP grading system was based on Gleason score according to Zhao, 2019a [89].

^4^Endpoint included biochemical recurrence defined as serum PSA ≥ 0.2 ng/ml after treatment, clinical metastasis or PCa-specific death.

^5^PFS defined as time to development of CRPC from initiation of ADT where progression to CRPC was defined as three consecutive monthly increases in serum PSA level against ADT according to Guan, 2016 [42].

^6^Biochemical recurrence defined as serum PSA ≥ 0.2 ng/ml after treatment.

^7^Biochemical recurrence defined as serum PSA ≥ 0.4 ng/ml after treatment.

^8^PFS defined as time to radiological/clinical progression from initiation of ARTA according to Sharova, 2021 [78].

**Table 3:** Allocation of 11 studies into 4 meta-analyses

Outcome	Handling of miR-21 data	No. of studies	Total no. of participants	Study IDs	Analysis
**RFS**	Dichotomous	4	838	Amankwah, 2013 [31]; Leite, 2015 [60]; Li, 2012 [61]; Melbø-Jørgensen, 2014 [68]	1
	Continuous	2	255	Zedan, 2017 [85]; Zhao, 2019a [89]	2
**OS**	Dichotomous	4	307	Lin, 2014 [64]; Lin, 2017 [65]; Sharova, 2021 [78]; Yang, 2016 [84]	3
**PFS**	Dichotomous	2	116	Guan, 2016 [42]; Sharova, 2021 [78]	4

Eleven eligible studies were allocated into four separate meta-analyses according to outcomes and handlings of miR-21 data. Note: Sharova, 2021 [78] with two outcomes was allocated into Analyses 3 and 4.

Abbreviations: OS, overall survival; PFS, progression-free survival; RFS, recurrence-free survival.

For population ‘P’, two studies from the same research group (Lin, 2014 [64] and Lin, 2017 [65]) included male patients diagnosed with CRPC that underwent docetaxel chemotherapy (a different set of patients was used for each study, therefore no double counting). Participants of Guan, 2016 [42] and Sharova, 2021 [78] received androgen deprivation therapy (ADT) and androgen receptor-targeted agents (ARTA) respectively; However, Sharova, 2021 [78] only included metastatic castration-resistant prostate cancer (mCRPC) patients. The rest of the studies (*n*=7) included male PCa patients that underwent resection surgeries such as radical prostatectomy (RP) and/or regional lymph node dissection. Not all studies reported the age range of participants, but it is apparent from available information that they were all around middle to old age groups at baseline (≥40 years).

For index prognostic factor ‘I’, Lin, 2014 [64]; Lin, 2017 [65]; Sharova, 2021 [78] and Yang, 2016 [84] measured circulating miR-21 in plasma, serum or peripheral blood mononuclear cell (PBMC) samples while the rest (*n*=7) measured tissue miR-21 in formalin-fixed paraffin-embedded (FFPE) tumor samples; Li, 2012 [61] and Zedan, 2017 [85] measured miR-21 level by *in situ* hybridization (ISH) methods that are semi-quantitative, while the rest (*n*=9) used real-time quantitative polymerase chain reaction (RT-qPCR) techniques that are highly sensitive and specific [93].

For comparator prognostic factors ‘C’, the most frequently included ones were Gleason score/grade (GS/GG; *n*=10 except Lin, 2017 [65]), PSA (*n*=10 except Amankwah, 2013 [31]) and pathological/clinical stage (pT/cT; *n*=8 except Lin, 2014 [64], Lin, 2017 [65] and Sharova, 2021 [78]). These were followed by age (*n*=6), hemoglobin (*n*=3), surgical margin (*n*=3), lymph node metastasis (pN; *n*=2) and alkaline phosphatase (*n*=2). Body mass index (BMI), capsular invasion, visceral metastasis, perineural infiltration, tumor size, vascular infiltration, digital rectal examination (DRE), prostate volume, neutrophil/lymphocyte ratio and time to CRPC were each included once between six studies (Amankwah, 2013 [31]; Li, 2012 [61]; Lin, 2014 [64]; Melbø-Jørgensen, 2014 [68]; Sharova, 2021 [78]; Zhao, 2019a [89]).

For outcomes of interest ‘O’, Lin, 2014 [64]; Lin, 2017 [65]; Sharova, 2021 [78] and Yang, 2016 [84] observed for overall survival (OS) defined as time from the date of treatment to the date of death; Guan, 2016 [42] and Sharova, 2021 [78] observed for progression-free survival (PFS), defined as time to development of CRPC from initiation of ADT by Guan, 2016 [42], and as time to radiological/clinical progression from initiation of ARTA by Sharova, 2021 [78]. The rest (*n*=6) observed for recurrence-free survival (RFS), generally defined as time from the date of treatment to the date of biochemical recurrence (BCR) with slight variations as indicated in Table 2 footnotes *d, f* and *g*. Latest follow-up times across studies ranged from 45 months (Lin, 2017 [65]) to 254 months (Amankwah, 2013 [31]), averaging up to 125 months (∼10 years). Not enough information was provided in Zedan, 2017 [85] to estimate the follow-up period.

### Risk of bias within studies

Risk of bias within each eligible study was assessed using the QUIPS tool [26]; two independent judgments were made before reaching consensus. Final ratings of risk of bias within the 11 studies eligible for meta-analyses are summarized in Table 4.

**Table 4:** Risk of bias within studies assessed using QUIPS tool

Study ID	QUIPS domains					
	1 Study participation	2 Study attrition	3 Prognostic factor measurement	4 Outcome measurement	5 Adjustment for covariates	6 Statistical analysis and reporting
Amankwah, 2013 [31]	HIGH	LOW	LOW	LOW	LOW	LOW
Guan, 2016 [42]	UNCLEAR	LOW	LOW	LOW	HIGH	LOW
Leite, 2015 [60]	UNCLEAR	LOW	LOW	LOW	LOW	HIGH
Li, 2012 [61]	UNCLEAR	LOW	MODERATE	LOW	LOW	HIGH
Lin, 2014 [64]	UNCLEAR	LOW	LOW	LOW	HIGH	MODERATE
Lin, 2017 [65]	UNCLEAR	LOW	LOW	LOW	HIGH	MODERATE
Melbø-Jørgensen, 2014 [68]	LOW	LOW	LOW	LOW	LOW	MODERATE
Sharova, 2021 [78]	MODERATE	LOW	LOW	LOW	HIGH	LOW
Yang, 2016 [84]	UNCLEAR	MODERATE	LOW	LOW	LOW	UNCLEAR
Zedan, 2017 [85]	MODERATE	LOW	MODERATE	LOW	UNCLEAR	UNCLEAR
Zhao, 2019a [89]	LOW	LOW	LOW	LOW	HIGH	LOW

Overall, no eligible study achieved LOW risk of bias in all domains. Most concerns in risk of bias were around domain 5 and 6 mainly due to inadequate adjustment for predefined important prognostic factors and selective reporting. The lack of rationale for sample size appears to be a common problem across the majority of eligible studies.

### Meta-analyses and sensitivity analyses

For all outcomes, results of each study eligible for meta-analyses are summarized in Table 5 (*n*=11). Six studies observed RFS, four observed OS, and two observed PFS. Effect estimates were pooled as HR (95% CI) based on fixed-effect inverse variance method. Statistical heterogeneity was determined by visual inspection of the forest plot, Chi^2^ test and *I*^2^ test (Chi^2^ *P*≤0.1 indicates significant heterogeneity; *I*^2^ < 30% denotes low/unimportant heterogeneity, 30–60% moderate heterogeneity, 50–90% substantial heterogeneity and 75–100% considerable heterogeneity).

**Table 5:** Summary of results of individual studies eligible for meta-analysis

Outcome (Analysis)	Study ID	Event /Total	Univariate analysis: Unadjusted HR (95% CI)	Multivariate analysis: Adjusted HR (95% CI)	Covariates adjusted for^*^
**RFS (1)**	Amankwah, 2013 [31]	28/65 (43%)	(Cut-off = median; log-rank *P*<0.0001) KM plot favouring high miR-21 Estimated HR (95% CI)^1^: = 4.83 (2.26–10.35), *P*=0.00005 Inverse^2^: = 0.21 (0.10–0.44), *P*=0.00005	1.99 (0.70–5.64), *P*=0.20 Inverse^2^: = 0.50 (0.18–1.42), *P*=0.20	Age GS cT
	Leite, 2015 [60]	50/127 (39%)	(Cut-off = median; log-rank *P*=0.003) KM plot favoring low miR-21 Estimated HR (95% CI)^1^: = 2.32 (1.33–4.03), *P*=0.003	2.505 (1.356–4.629), *P*=0.003	GG PSA pT
	Li, 2012 [61]	116/168 (69%)	(Cut-off = median; log-rank *P*<0.001) KM plot favoring low miR-21 Estimated HR (95% CI)^1^: = 1.91 (1.33–2.75), *P*=0.0005	2.059 (1.075–3.944), *P*=0.029	Age Capsular invasion GS pN PSA pT Surgical margin
	Melbø-Jørgensen, 2014 [68]	170/478 (36%)	(Cut-off = 4^th^ quartile; log-rank *P*=0.006) KM plot favoring low miR-21 Estimated HR (95% CI)^1^: = 1.65 (1.15–2.36), *P*=0.006	1.4 (1.0–1.9), *P*=0.089	Apical PSM GG Non-apical PSM Perineural infiltration PSA pT Vascular infiltration
**RFS (2)**	Zedan, 2017 [85]	19/49 (39%)	(Continuous miR-21) 1.231 (0.697–2.177), *P*=0.474	*(No multivariate analysis data)*	*(N/A)*
	Zhao, 2019a [89]	98/206 (48%)	(Continuous miR-21) 1.12 (1.01–1.24), *P*=0.049	(Continuous miR-21) 1.35 (0.86–2.12), *P*=0.188	15 other miRNAs of interest
**OS (3)**	Lin, 2014 [64]	55/97 (57%)	(High vs. low miR-21, cut-off = median) 2.3 (1.3–3.9), log-rank *P*=0.004	*(No multivariate analysis data)*	*(N/A)*
	Lin, 2017 [65]	53/87 (61%)	(High vs. low miR-21, cut-off = median) 1.2204 (0.7028–2.1192), *P*=0.477	(Continuous miR-21) 1.1488 (0.8849–1.4916), *P*=0.303	Alkaline phosphatase Hemoglobin PSA
	Sharova, 2021 [78]	13/31 (42%)	(Cut-off = 2.69; log-rank p = 0.0067) KM plot favoring high miR-21 5.2 (1.7–15.7), *P*=0.0191 Inverse^2^: = 0.192 (0.064–0.588), *P*=0.0191	5.8 (1.0–33.1), *P*=0.049 Inverse^2^: = 0.172 (0.03–1.0), *P*=0.049	Hemoglobin Time to CRPC
	Yang, 2016 [84]	42/92 (46%)	(Cut-off not stated; log-rank *P*<0.05) KM plot favoring low miR-21 Estimated HR (95% CI)^1^: = 2.02 (1.09–3.73), *P*=0.025	3.567 (1.287–9.882), *P*=0.014	Age BCR Bone metastasis cT GS PSA
**PFS (4)**	Guan, 2016 [42]	47/85 (55%)	(Cut-off = mean; log-rank *P*=0.006) KM plot favoring low miR-21 2.381 (1.250–4.537), *P*=0.008	1.985 (1.032–3.817), *P*=0.040	cT
	Sharova, 2021 [78]	26/31 (84%)	(Cut-off = 2.69; log-rank *P*= =0.0002) KM plot favoring high miR-21 7.4 (2.6–21.2), *P*=0.0021 Inverse^2^: = 0.135 (0.047–0.385), *P*=0.0021	4.8 (1.3–17.8), *P*=0.019 Inverse^2^: = 0.208 (0.056–0.769), *P*=0.019	Hemoglobin Time to CRPC

Abbreviations: BCR, biochemical recurrence; CI, confidence interval; CRPC, castration-resistant prostate cancer; cT, clinical tumor stage; GG, Gleason grade; GS, Gleason score; HR, hazard ratio; KM, Kaplan–Meier; N/A, not applicable; OS, overall survival; PFS, progression-free survival; pN, lymph node metastasis; PSA, prostate-specific antigen; PSM, positive surgical margins; pT, pathological tumor stage; RFS, recurrence-free survival.

* GS/GG and pT/cT were predefined as important prognostic factors that should be adjusted for in multivariate analysis.

^1^Unadjusted HR (95% CI) was not reported; hence it was estimated using an Excel calculator [94].

^2^The direction of effect estimates in Amankwah, 2013 [31] and Sharova, 2021 [78] were opposite to the rest of eligible studies; hence, they were inverted (i.e. divided by 1) to obtain the complementary value.

#### Analysis 1: Recurrence-free survival; dichotomous miR-21 data (n=4)

**Figure 2:**
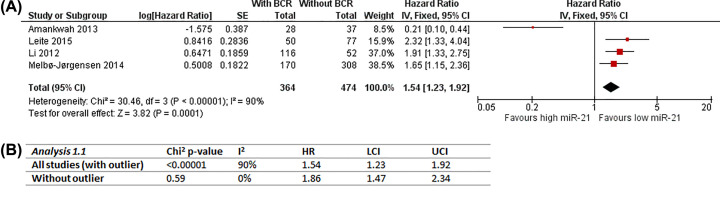
Analysis 1.1: Meta-analysis of dichotomous miR-21 expression with recurrence-free survival (unadjusted) (**A**) Unadjusted results and forest plot, RevMan5.4 snapshot. (**B**) Sensitivity analysis of impact of outlier (Amankwah, 2013 [31]); BCR, biochemical recurrence; CI, confidence interval; HR, hazard ratio; IV, inverse variance; LCI, lower confidence interval; SE, standard error; UCI, upper confidence interval.

**Figure 3:**
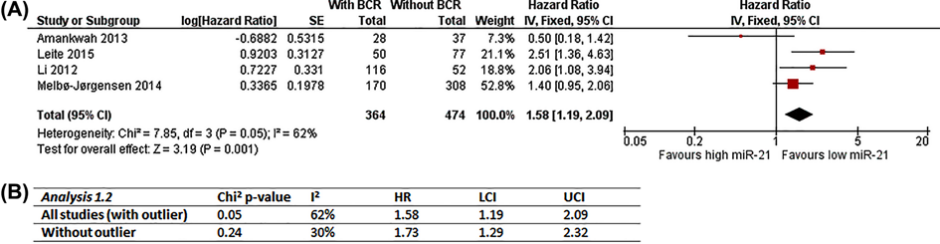
Analysis 1.2: Meta-analysis of dichotomous miR-21 expression with recurrence-free survival (adjusted) (**A**) Adjusted results and forest plot, RevMan5.4 snapshot. (**B**) Sensitivity analysis of impact of outlier (Amankwah, 2013 [31]); BCR, biochemical recurrence; CI, confidence interval; HR, hazard ratio; IV, inverse variance; LCI, lower confidence interval; SE, standard error; UCI, upper confidence interval.

This analysis includes Amankwah, 2013 [31]; Leite, 2015 [60]; Leite, 2012 [61] and Melbø-Jørgensen, 2014 [68] as they have observed RFS as outcome and dichotomised tissue miR-21 expression data into high and low groups (median as cut-off for Amankwah, 2013 [31]; Leite, 2015 [60] and Li, 2012 [61]; 4^th^ quartile for Melbø-Jørgensen, 2014 [68]). Unadjusted and adjusted effect estimates of all four studies were combined in Analysis 1.1 (Figure 2A) and Analysis 1.2 (Figure 3A) respectively for comparison to examine the effect of heterogeneity caused by differences in covariate adjustment. Overall number of participants is 838 (364 with BCR; 474 without BCR).

The overall effect of unadjusted estimates, as shown in the forest plot of Analysis 1.1, favors low miR-21, suggesting high miR-21 expression is associated with higher risk of BCR (HR = 1.54, 95% CI = 1.23–1.92). Statistical heterogeneity tests indicate significantly considerable heterogeneity (Chi^2^ *P*<0.00001; *I*^2^ = 90%), most likely caused by the presence of an outlier (Amankwah, 2013 [31]) which showed an opposite direction of effect estimate to the other studies. To probe this further, the impact of the outlier on this meta-analysis was assessed by sensitivity analysis. Results of sensitivity analysis (Figure 2B) confirmed the data from Amankwah, 2013 [31] as the source of statistical heterogeneity (*I*^2^ = 0% without outlier). However, the inclusion of the outlier did not change the effect estimate significantly; therefore, the results of Analysis 1.1 are still valid.

The overall effect of adjusted estimates (Analysis 1.2) is very close to that of unadjusted estimates (Analysis 1.1) supporting the same conclusion, i.e., it favors low miR-21, suggesting high miR-21 expression is associated with higher risk of BCR (HR = 1.58, 95% CI = 1.19–2.09; Figure 3A). However, different from Analysis 1.1, Melbø-Jørgensen, 2014 [68] now occupied over half of the overall weight (52.8%) with Li, 2012 [61] weighing only 18.8%. Amankwah, 2013 [31] still appears to be outlying, and statistical heterogeneity tests also indicate significantly substantial heterogeneity (Chi^2^ *P*=0.05; *I*^2^ = 62%). Again, sensitivity analysis repeating Analysis 1.2 without Amankwah, 2013 [31] reduced statistical heterogeneity to insignificant and low/unimportant (*I*^2^ = 30%; Figure 3B), verifying the outlying estimate as the source of statistical heterogeneity. The slight difference in overall effect reveals that the inclusion of the outlier has limited impact, and that the results of Analysis 1.2 are robust.

Comparing the two analyses, covariate adjustment in Analysis 1.2 had brought Amankwah, 2013 [31] closer to the other studies with the upper CI arm crossing the line of no effect and overlapping with others’ that might explain the lower statistical heterogeneity indicated by *I*^2^ values compared to Analysis 1.1 (62% vs. 90%). However, eliminating the effect of outlier, higher *I*^2^ value of adjusted estimates compared with unadjusted (30% vs. 0%) implies that differences in covariate adjustment might have introduced some heterogeneity, though low and insignificant.

#### Analysis 2: Recurrence-free survival; continuous miR-21 data (n=2)

**Figure 4:**
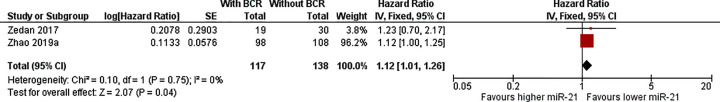
Analysis 2: Meta-analysis of continuous miR-21 expression with recurrence-free survival Unadjusted results and forest plot, RevMan5.4 snapshot; BCR, biochemical recurrence; CI, confidence interval; HR, hazard ratio; IV, inverse variance; SE, standard error.

This analysis includes Zedan, 2017 [85] and Zhao, 2019a [89] as both have observed RFS as outcome against continuous miR-21 expression in tissue samples. Only unadjusted effect estimates were combined in Analysis 2 (Figure 4) because of lack of multivariate analysis data for Zedan, 2017 [85]. Overall number of participants is 255 (117 with BCR; 138 without BCR).

The overall effect estimate (HR = 1.12, 95% CI = 1.01–1.26) favors lower miR-21, indicating that higher miR-21 expression is associated with higher risk of BCR. The overall effect in the forest plot showed high precision from the tight CI and statistical heterogeneity is very low (Chi^2^ *P*=0.75; *I*^2^ = 0%). However, the data points are very close to the line of no effect with the lower CI of Zedan, 2017 [85] across. The overall weight is dominated by Zhao, 2019a [89] (96.2%) between only two studies.

#### Analysis 3: Overall survival; dichotomous miR-21 data (n=4)

**Figure 5:**
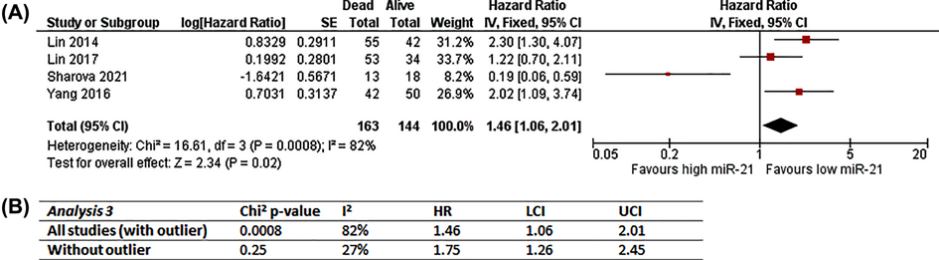
Analysis 3: Meta-analysis of miR-21 expression with overall survival (**A**) Unadjusted results and forest plot, RevMan5.4 snapshot. (**B**) Sensitivity analysis of impact of outlier (Sharova, 2021 [78]); CI, confidence interval; HR, hazard ratio; IV, inverse variance; LCI, lower confidence interval; SE, standard error; UCI, upper confidence interval.

This analysis included Lin, 2014 [64]; Lin, 2017 [65]; Sharova, 2021 [78] and Yang, 2016 [84] as they are similar in outcome observed (OS), handling of miR-21 data (dichotomised) and source of miR-21 (circulating samples). Only unadjusted effect estimates were combined in Analysis 3 (Figure 5A) because of lack of multivariate analysis data for Lin, 2014 [64] and differences in covariate adjustment and handling of miR-21 data in multivariate analysis for Lin, 2017 [65]. Overall number of participants is 307 (163 dead; 144 alive).

The overall effect in Analysis 3 favors low miR-21, suggesting high miR-21 expression is associated with higher risk of death (HR = 1.46, 95% CI = 1.06–2.01; Figure 5A). Sharova, 2021 [78] was outlying in the opposite direction to the rest and mostly likely have caused the considerable heterogeneity (Chi^2^ *P*=0.0008; *I*^2^ = 82%); Therefore the impact of including Sharova, 2021 [78] in Analysis 3 was examined in sensitivity analysis (Figure 5B). Sensitivity analysis repeating Analysis 3 without Sharova2021 [78] significantly reduced heterogeneity to low/unimportant level (Chi^2^ *P*=0.25; *I*^2^ = 27%; Figure 5B), confirming an outlier as the main source of heterogeneity, and that had brought the overall effect estimate closer to the line of no effect.

#### Analysis 4: Progress-free survival; dichotomous miR-21 data (n=2)

**Figure 6:**
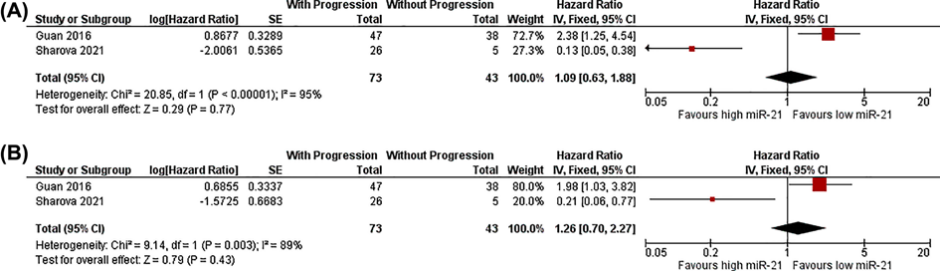
Meta-analyses of miR-21 expression with progression-free survival (**A**) Analysis 4.1: Unadjusted results and forest plot, RevMan5.4 snapshot. (**B**) Analysis 4.2: Adjusted results and forest plot, RevMan5.4 snapshot; CI, confidence interval; HR, hazard ratio; IV, inverse variance; SE, standard error.

Analysis 4 included Guan, 2016 [42] and Sharova, 2021 [78] because both studies observed PFS as outcome. Overall number of participants is 116 (73 with progression; 43 without progression). Figure 6A,B showed meta-analysis results along with forest plots of combined unadjusted and adjusted effect estimates respectively (Analyses 4.1 and 4.2). Neither analysis reached a significant overall effect (CIs crossing line of no effect), most likely since only two studies with opposite effect estimates were available, which also contributed to considerable heterogeneities (Chi^2^<0.1; *I*^2^>80%). Therefore, no meaningful conclusion could be drawn from Analysis 4.

### Qualitative summary and associations

Most of the 64 studies included in this review compared the association of miR-21 with commonly used clinicopathological prognostic factors (Table 6). These included Gleason score/grade (*n*=28), pathological/clinical stage (*n*=18), serum PSA level (*n*=18), risk stratification (*n*=12) and age at diagnosis (*n*=9). Association of miR-21 expression with recurrence (*n*=19) and metastasis (*n*=14) were also examined in many included studies. A few studies have compared miR-21 levels in/with prostate volume (*n*=4), chem-response (*n*=3), digital rectal examination (DRE) result (*n*=3), ethnicity (*n*=2) and surgical margin (*n*=2). Other comparisons made include genitourinary radiotoxicity (Kopcalic, 2019 [53]), neuroendocrine-like versus Adeno PCa (Ostano, 2020 [71]), follow-up time, family history (Shen, 2012 [79]) and reclassification (Zhao, 2019b [90]).

**Table 6:** Summary of association results of included studies

Association result			Gleason (*n*=28)^*^	Stage (*n*=18)	PSA (*n*=18)^*^	
***P*<0.05**	**Pos**	**T**	Arisan, 2020; **Guan, 2016**; **Li, 2012**; **Melbø-Jørgensen, 2014**; **Zhao, 2019a**	**Li, 2012**; **Melbø-Jørgensen, 2014**; Reis, 2012; **Zhao, 2019a**		
		**C**	Al-Qatati, 2017; Gurbuz, 2020; Ibrahim, 2019a; Ibrahim, 2019b; Ju, 2019; **Yang, 2016**	Al-Qatati, 2017; Gurbuz, 2020; Huang, 2015b; Ibrahim, 2019a; Ibrahim, 2019b; Ju, 2019; Stuopelyte, 2016; **Yang, 2016**	Al-Qatati, 2017; Gurbuz, 2020; Ibrahim, 2019b	
		**U**	Samaan, 2014			
	**Neg**	**T**	Ren, 2014	Ren, 2014		
***P*** **>0.05**	**Pos**	**T**	Katz, 2014; Kurul, 2019; Lichner, 2015; Reis, 2012; **Zedan, 2017**; Zedan, 2018	**Zedan, 2017**	**Li, 2012**; Reis, 2012; Zedan, 2018; **Zhao, 2019a**	
		**C**	Shen, 2012	Shen, 2012 Zedan, 2019	Ju, 2019; Shen, 2012; Zedan, 2018; Zedan, 2019	
	**Neg**	**T**	Kristensen, 2016^**^	Katz, 2014	**Zedan, 2017**	
		**C**	Kotb, 2014; Zedan, 2018; Zedan, 2019		**Sharova, 2021; Yang, 2016**; Zhao, 2019b	
	**No diff**	**T**	**Amankwah, 2013**		**Guan, 2016**; Katz, 2014	
		**C**	Farran, 2018; Foj, 2017; Stuopelyte, 2016			
**No** ***P*-value**	**Pos**	**T**		Hart, 2014		
	**No corr**	**C**			Agaoglu, 2011	

Association result			Recurrence (*n*=19)	Metastasis (*n*=14)	Risk (*n*=12)	Age (*n*=9)
***P*<0.05**	**Pos**	**T**	Leite, 2015; Li, 2012; Melbø-Jørgensen, 2014; Reis, 2012	Guan, 2016; Li, 2012	Zhu, 2019	
		**C**	Huang, 2015b; Ju, 2019; Yang, 2016	Agaoglu, 2011; Brase, 2011; Huang, 2015b; Ibrahim, 2019a; Ibrahim, 2019b; Watahiki, 2013; Yang, 2016; Ju, 2019	Foj, 2017; Ju, 2019; Shen, 2012	Zedan, 2019
	**Neg**	**T**	Suer, 2019^**^; Amankwah, 2013	Ren, 2014		Ren, 2014
		**C**		Danarto, 2020		
***P*>0.05**	**Pos**	**T**	Kurul, 2019; Leite, 2011; Ren, 2014		Katz, 2014; Leite, 2013; Zedan, 2017	
		**C**	Stuopelyte, 2016		Al-Qatati, 2017; Hoey, 2019; Sapre, 2014; Zedan, 2019	Huang, 2015b; Yang, 2016
	**Neg**	**T**	Katz, 2014	Leite, 2011	Lichner, 2013	Zhao, 2019a; Li, 2012
		**C**	Selth, 2013; Shen, 2012			Shen, 2012; Zhao, 2019b
	**No diff/corr**	**T**	Kristensen, 2016^**^; Zheng, 2014			Guan, 2016
		**C**	Singh, 2014			
**No** ***P*-value**	**Pos**	**T**		Bonci, 2016		

Most of the 64 studies included in this review compared the association of miR-21 with commonly used clinicopathological prognostic factors (Gleason score/grade; pathological/clinical stage; serum PSA level; risk stratification; age at diagnosis), as well as recurrence and metastasis.

Study IDs in **bold** were eligible for meta-analysis (*n*=11).

Possible part overlap of participants between Ibrahim, 2019a [48] and Ibrahim, 2019b [49].

Abbreviations: C, circulating miR-21; corr, correlation; diff, difference; Neg, negative association; Pos, positive association; PSA, prostate-specific antigen; T, tissue miR-21; U, unknown miR-21 source

* Zedan, 2018 [86] was counted twice as both tissue and plasma miR-21 expressions were measured.

** 3p strand of miR-21 was measured.

Results were grouped according to statistical significance (*P*<0.05/*P*>0.05), association direction (positive/negative) and sample source (tissue/circulating). Association measures varied between studies, these include fold change (FC), mean difference and correlation, meaning it was impractical to summarize findings according to comparison methods. Therefore, findings were summarised according to association directions. When higher miR-21 expression was associated with higher degree/presence of the comparators it was indicated as positive; when it was associated with lower degree/absence of the comparators it was negative.

Additional figures demonstrating association results can be found in Supplementary Figure SF 1A–G. Twelve out of 28 studies (43%) that compared miR-21 levels in different Gleason scores/grades found significant positive association of miR-21 levels from tissue and circulating samples. Twelve out of 18 studies (67%) that compared miR-21 levels in different pathological/clinical stages found significant positive association of miR-21 mostly from circulating samples as well as tissue. In contrast, only three studies reported significant positive association in circulating miR-21 and serum PSA. Seven out of 19 studies (37%) found significant positive association between tissue/circulating miR-21 and biochemical recurrence, defined generally as biochemical recurrence determined by rise in serum PSA ≥ 0.2–0.4 ng/ml after treatment. Ten out of 14 studies (71%) that compared miR-21 levels in samples of metastatic versus localized PCa patients found significant positive association between metastatic PCa and miR-21 mostly in circulating samples (*n*=8; tissue *n*=2). 11 out of 12 studies (92%) that examined risk stratification reported positive association of higher risk with elevated miR-21 expression, although only 4 (33%) of these were found to be statistically significant.

### Certainty of evidence – GRADE

Publication bias was not assessed due to low number of studies eligible for each analysis. No analysis was rated up for large effect, dose response or plausible confounding. Table 7 presented judgments of rate-downs and overall certainties of each analysis. Overall certainty is MODERATE for Analysis 1.2; LOW for Analyses 1.1 and 2; VERY LOW for Analyses 3, 4.1 and 4.2. See Supplementary Table ST 7 for full rationales for rating down certainty of evidence.

**Table 7:** Certainty of evidence in each analysis (GRADE)

Analysis	Outcome	Pooled result HR (95% CI)	No. of participants	Certainty rate-downs	Overall certainty^5^
1.1	RFS^1,3^	1.54 (1.23–1.92)	838 (4 studies)	- RoB: High RoB in 3 studies - Imprecision: Estimated HR in all studies	LOW
1.2	RFS^2,3^	1.58 (1.19–2.09)	838 (4 studies)	- RoB: High RoB in 3 studies	MODERATE
2	RFS^1,4^	1.12 (1.01–1.26)	255(2 studies)	- RoB: Unadjusted HR & high RoB in 1 study - Imprecision: CI close to HR 1	LOW
3	OS^1,3^	1.46 (1.06–2.01)	307 (4 studies)	- RoB: Unadjusted HR & high RoB in 3 studies - Indirectness: Lin 2014 & Lin 2017 recruited CRPC patients to address chemo-response - Imprecision: Estimated HR in 1 study; CI close to HR 1	VERY LOW
4.1	PFS^1,3^	1.09 (0.63–1.88)	116 (2 studies)	- RoB: High RoB in both studies - Inconsistency: Opposite direction results - Imprecision: Wide CI crossing HR 1	VERY LOW
4.2	PFS^2,3^	1.26 (0.70–2.27)	116 (2 studies)	- RoB: High RoB in both studies - Inconsistency: Opposite direction results - Imprecision: Wide CI crossing HR 1	VERY LOW

**Abbreviations:** CI, confidence interval; CRPC, castration-resistant prostate cancer; HR, hazard ratio; OS, overall survival; RFS, recurrence-free survival; RoB, risk of bias.

^1^Unadjusted effect estimates.

^2^Adjusted effect estimates.

^3^Dichotomised miR-21 levels.

^4^Continuous miR-21 levels.

^5^HIGH: We are very confident that the variation in risk associated with miR-21 expression lies close to that of the estimate; MODERATE: We are moderately confident that the variation in risk associated with miR-21 expression is likely to be close to the estimate, but substantial difference is possible; LOW: We have limited certainty in the estimate, the variation in risk associated with miR-21 expression may be substantially different from the estimate; VERY LOW: We have very little certainty in the estimate, the variation in risk associated with miR-21 expression is likely to be substantially different from the estimate (GRADE [28]).

## Discussion

In this report, we have performed the first systematic review and meta-analysis of miR-21 as a prognostic factor in PCa. miR-21 is one of the most studied miRNAs in cancer and has been shown to play a role in many different cellular mechanisms which can contribute to cancer progression, including PCa [95]. Although miR-21 targets many genes and thus regulates many genetic pathways, it appears to act in a primarily oncogenic fashion with many studies reporting elevated levels in samples taken from cancer patients. Despite this body of evidence, there is still doubt about whether it may be a useful biomarker for cancer prognosis, so robust analyses of existing studies are needed to determine its value for clinical application and to inform the optimal design of future studies.

The pooled results of all meta-analyses reported here supported an association between high miR-21 expression and poor prognosis in PCa. Regarding RFS, Analysis 1.2 estimated a 58% increased risk of BCR for high baseline expression of tissue miR-21 (HR = 1.58, 95% CI = 1.19–2.09) with MODERATE certainty of evidence. For OS, Analysis 3 estimated a 75% increased risk of death for high baseline expression of circulating miR-21 with VERY LOW certainty of evidence (HR = 1.75, 95% CI = 1.26–2.45). No meaningful conclusion could be drawn for PFS in Analysis 4 due to considerable heterogeneity between only two eligible studies. The heterogeneity could be attributed to differences in population, miR-21 source and PFS definition. Guan, 2016 [42] recruited pathologically confirmed PCa patients while Sharova, 2021 [78] only included mCRPC patients; Guan, 2016 [42] detected miR-21 from FFPE tissue samples while Sharova, 2021 [78] examined it in plasma samples; Guan, 2016 [42] defined PFS as time to development of CRPC while Sharova, 2021 [78] defined it as time to radiological/clinical progression. Analysis 4 demonstrated the importance of only combining results of similar studies as a basic principle of meta-analysis. The limited certainty in OS result and lack of similar studies in PFS for a meaningful meta-analysis indicated that more high-quality prognostic studies are needed for OS and PFS. Nevertheless, our systematic approach and meta-analyses found consistent evidence that miR-21 may have prognostic value in PCa. These data suggest miR-21 can be put forward as a strong candidate for the prognosis of the disease, although further work is clearly needed to prove its value more conclusively as a biomarker.

Our results agreed with systematic reviews in other cancers such as non-small cell lung, pancreatic and colorectal cancers [96–98]. These suggested high tissue miR-21 as an unfavourable prognostic biomarker. Circulating miR-21 overexpression was also associated with poor prognosis in digestive system and breast cancers [18,19,99]. This is not unexpected, given that it is generally agreed to act as an oncogene, but this understanding of its functional role in the cell can only be translated into medical application when the literature available is subject to methodical evaluation in studies such as these.

However, it is worth noting that the authors of the papers subject to meta-analysis here all indicated limitations with their studies. We recorded this as part of our data gathering process and further probed it through our quality assessment of individual studies. Pooled evidence by QUIPS and GRADE methodologies revealed sources of risk of bias and down-rate of certainty of evidence. In several studies, selective reporting and failure to adjust for the core set of covariates increased risk of bias and imprecision, thus decreased certainty of evidence. Furthermore, publication bias could not be properly assessed due to inadequate number of studies included in individual analysis. This was mainly due to high heterogeneity across studies, such as differences in outcome, handling of miR-21 data and sample source. The limited similarities meant that eligible studies had to be split into separate small analyses, therefore reducing the impact of meta-analyses. It was unfortunate that so few of the published studies met the required criteria for inclusion in meta-analysis, which limits the strength of the analyses and our subsequent ability to draw firm conclusions. Although the very nature of a properly conducted meta-analysis is to be robust and consistent in the application of the methodology, limitations in selected studies are inevitably reflected in the limitations of the subsequent meta-analyses, since the patient numbers and/or measured parameters are less than ideal. Perhaps that is to be expected since miRNAs as biomarkers is a relatively recent field of research, but it is clear that a lack of standardised approach to these type of biomarker studies makes it difficult to evaluate the clinical usefulness of miRNAs as prognostic biomarkers. Therefore, for any researchers carrying out future cancer prognostic studies of this type, it is highly recommended that they adhere to the Reporting Recommendations for Tumour Marker Prognostic Studies (REMARK) guidelines for proper study design, conduct, analysis and reporting [100]. This will reduce risk of bias and heterogeneity across studies to generate higher quality evidence and more opportunity for comparison in meta-analyses like the ones presented here. Evidently, Zhao, 2019a [89] was the only included study that followed the guidelines and achieved LOW risk of bias in most QUIPS domains.

Although several of the full-text studies reviewed were not eligible for meta-analysis, they nevertheless contained useful data about the association of miR-21 with PCa, which is important to discuss since it can inform future study design. Overall, several studies in this review supported the hypothesis that there is a significant positive association between miR-21 expression and various clinical measurements of PCa progression, such as stage, Gleason score, risk groups, metastasis and recurrence. Notably, very few studies found a significant association between miR-21 expression and serum PSA level or age at diagnosis.

However, for clinical application of miR-21 analysis, several barriers must be overcome. A standardized method for measuring miR-21 must be decided upon. RT-qPCR, as used in many of the studies reported here, would seem the most appropriate technique at present in terms of sensitivity and applicability. Nevertheless, agreement is needed on common normalisation approaches and comparable internal controls, such as reference genes. Even with these measures in place, a consensus would then be needed on an appropriate cut-off value for prognostic outcome, which was very variable in the studies evaluated here. Another important consideration is that the correct miR-21 strand is being measured, since there is no guarantee that expression of miR-21-3p and miR-21-5p will be similar. The majority of the studies in this review did not specify miR-21 strand, which is also another reason to be cautious about the interpretation of the results presented here.

Even if standardized approaches meant RT-qPCR was accepted as suitably sensitive and accurate method, the sample type in which to measure the miR-21 target is a further complication. Among 64 studies included in this review, 32 measured miR-21 levels in circulating samples, including plasma, serum, PBMC, urine, exosome and whole blood; 30 measured miR-21 levels in tissue samples; Zedan, 2018 [86] measured from both sample types; and Samaan, 2014 [74] did not clearly state the sample source. Zedan, 2018 [86] found significant correlation of miR-21 levels between matched tissue and plasma samples from 25 healthy patients (*r*=0.58, *P*<0.01) but not in 21 PCa patients (*P*=0.42). It is not certain that tissue and biofluid levels of miR-21 will be directly comparable, and it is also possible that different outcomes might be better predicted by miR-21 expression in one particular sample type. Thus, further inter- and intra-individual analyses would be needed to determine the relative value of these different sample types. It is therefore clear that for miR-21, or any other miRNA, to gain clinical acceptance as disease biomarker, it requires well-designed, prospective clinical studies to validate the findings reported here. Ideally, these studies should utilise the same PICOT criteria, ensuring common outcomes and measurements can then be compared between studies and across different research centres.

Nevertheless, even though there are not yet enough well-designed studies to conclusively prove biomarker potential of miRNAs, it does appear increasingly likely that they will be used in future as non-invasive, liquid biomarkers for cancer and other diseases [101,102]. With this in mind, miR-21 is a very attractive candidate to profile, since it is abundantly expressed in both tissue and biofluids, making it easy to measure [14,103]. In relation to PCa specifically, its involvement in promoting cancer growth, and related roles in important pathological changes, such as epithelial-to-mesenchymal transition (EMT), is now well established [14,104], so there is a strong biological rationale for measuring its expression as a marker of disease progression. It is worth remembering however that miRNAs often work synergistically as a regulatory network for gene expression, so the involvement of miR-21 with other miRNAs should be considered. For instance, while this paper was being prepared, another systematic review and meta-analysis was published which reported the prognostic significance of 15 microRNAs related to metastasis and EMT process in PCa patients [105]. Surprisingly, miR-21 was not included among them, but the authors did acknowledge the link between their selected miRNAs and miR-21 in their discussion, and they concluded that a miRNA panel of biomarkers would be optimal to determine progression risk. Similarly, another recent paper used meta-analysis methods to identify miR-21 as one of several miRNAs which could predict response to ADT [106]. Profiling different miRNAs in parallel makes sense, since many miRNAs are known to be involved in PCa development [101,103]. It is also unlikely that miR-21 (or any other miRNA) as a single biomarker would be sufficient to accurately predict any given patient outcome. Therefore, the ability to measure expression levels of other miRNAs, or other genetic parameters, in combination with miR-21 should be built into the design of future studies investigating its prognostic value in cancer A multivariate profiling approach to PCa prognosis, which includes measurement of miR-21, would be a sensible approach to take.

## Conclusion

Meta-analyses of 11 studies in this report showed that high miR-21 expression was associated with poor prognosis in PCa. Qualitative summary of all 64 studies also found positive association of miR-21 expression with various prognostic factors for PCa. These findings corroborate data from other systematic reviews which have shown similar findings for miR-21 in various cancers. However, further research is needed, including more high-quality investigations that follow standardized guidelines for study design. With continued effort, miR-21 could prove to be a clinically useful prognostic biomarker in prostate cancer.

## Supplementary Material

Supplementary Figure S1 and Tables S1-S7

## Data Availability

The datasets analyzed in the present study are available from the published papers that have been cited in this manuscript.

## Abbreviations

BCR: biochemical recurrence
CI: confidence interval
CRPC: castration-resistant prostate cancer
EMT: epithelial-to-mesenchymal transition
FC: fold change
HR: hazard ratio
IV: inverse variance
LCI: lower confidence interval
OS: overall survival
RFS: recurrence-free survival
RoB: risk of bias
UCI: upper confidence interval
