# Supplementary material for: Prognostic value of miR-21 for prostate cancer: a systematic review and meta-analysis
Source: Biosci Rep. 2022 Jan 11;42(1):BSR20211972. doi: 10.1042/BSR20211972 (PMC8753345; doi:10.1042/BSR20211972)
Supplement: Supplementary Figure S1 and Tables S1-S7 [file BSR-2021-1972_supp.pdf]

SUPPLEMENTARY FIGURES

a

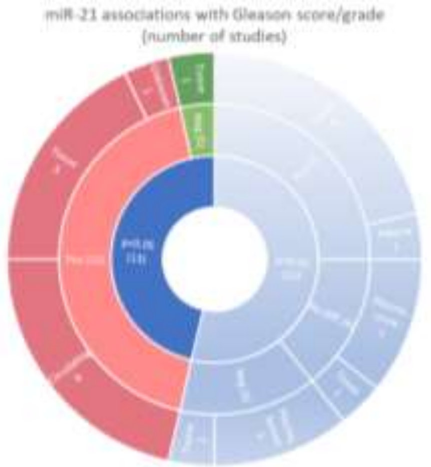

b

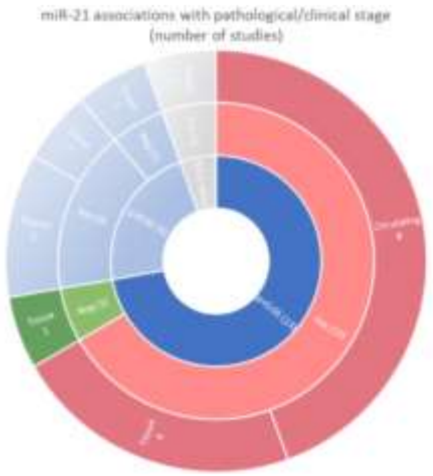

c

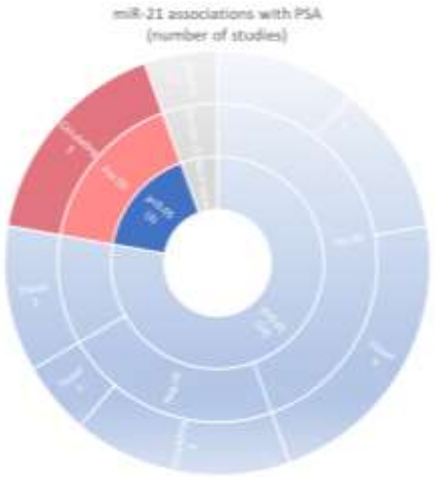

d

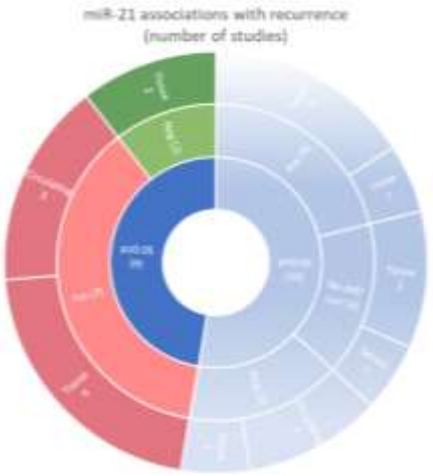

e

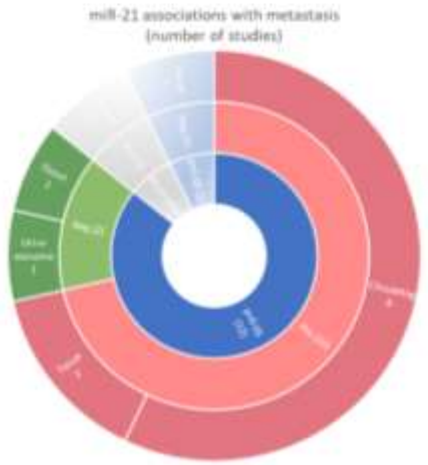

f

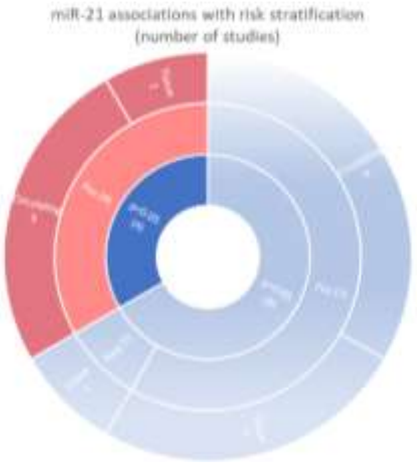

g

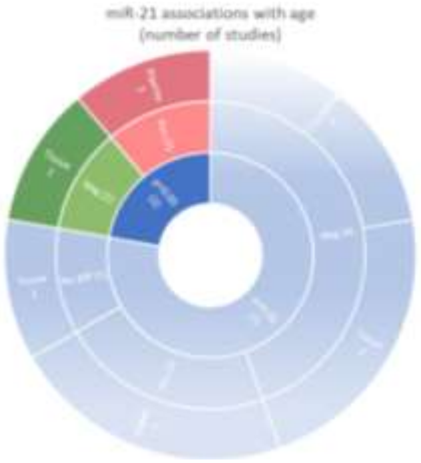

SF 1: Associations of miR-21 expression with clinicopathological measurements. (a) Gleason score/grade, (b) Stage, (c) PSA, (d) Recurrence, (e) Metastasis, (f) Risk stratification and (g) Age at diagnosis

ST 1: Search strategies in electronic databases

Medline (Ovid)

1. exp MicroRNAs/
2. (microRNA or miRNA or microRNA-21 or microRNA21 or miRNA-21 or miRNA21 or miR-21 or miR21)
3. exp Prostatic Neoplasms/
4. (prostat* cancer* or prostat* carcinoma* or prostat* tumo?r* or prostat* neoplasm* or prostat* adenocarcinoma* or PRAD)

5. exp Biomarkers/
6. exp Prognosis/
7. exp Survival Analysis/
8. (biomarker* or marker* or prognos* or survival)
9. 1 or 2
10. 3 or 4
11. 5 or 6 or 7 or 8
12. 9 and 10 and 11
13. limit 12 to yr="2010 -Current"
14. limit 13 to english language
15. limit 14 to (case reports or editorial or english abstract or letter or meta analysis or "review" or "systematic review")
16. 14 not 15

EMBASE

1. exp microRNA 21/
2. exp microRNA/
3. (microRNA or miRNA or microRNA-21 or microRNA21 or miRNA-21 or miRNA21 or miR-21 or miR21)
4. 1 or 3
5. exp prostate cancer/
6. (prostat* cancer* or prostat* carcinoma* or prostat* tumo?r* or prostat* neoplasm* or prostat* adenocarcinoma* or PRAD)

7. 5 or 6
8. exp prognosis/
9. exp biological marker/
10. exp survival/ or exp survival analysis/
11. (biomarker* or marker* or prognos* or survival)
12. 8 or 9 or 10 or 11
13. 4 and 7
14. 12 and 13
15. limit 14 to yr="2010 -Current"
16. limit 15 to english language
17. limit 16 to (meta analysis or "systematic review")
18. limit 16 to (books or chapter or conference abstract or editorial or letter or "review" or short survey)
19. 17 or 18
20. 16 not 19

Web of Science (Core Collection)

1. TOPIC: ("microRNA-21" OR "microRNA21" OR "miRNA-21" OR "miRNA21" OR "miR-21" OR "miR21" OR microRNA OR miRNA)
2. TOPIC: ("prostat* cancer*" or "prostat* carcinoma*" or "prostat* tumo?r*" or "prostat* neoplasm*" or "prostat* adenocarcinoma*" or PRAD)
3. TOPIC: (biomarker* or marker* or prognos* or survival)
4. #3 AND #2 AND #1 Refined by: [excluding] PUBLICATION YEARS: (2008 OR 2007 OR 2006 OR 2009) AND LANGUAGES: (ENGLISH) AND [excluding] DOCUMENT TYPES: (EDITORIAL MATERIAL OR LETTER OR REVIEW OR PROCEEDINGS PAPER OR RETRACTED PUBLICATION OR RETRACTION OR MEETING ABSTRACT OR BOOK CHAPTER)

Scopus

(TITLE-ABS-KEY (biomarker* OR marker* OR prognos* OR survival)) AND ((TITLE-ABS-KEY ("microRNA-21" OR "microRNA21" OR "miRNA-21" OR "miRNA21" OR "miR-21" OR "miR21" OR "circulating microRNA*")) AND (TITLE-ABS-KEY ("prostat* cancer*" OR "prostat* carcinoma*" OR "prostat* tumo?r*" OR "prostat* neoplasm*" OR "prostat* adenocarcinoma*" OR prad))) AND (EXCLUDE (PUBYEAR , 2009) OR EXCLUDE (PUBYEAR , 2008)) AND (LIMIT-TO (LANGUAGE , "English")) AND (EXCLUDE (DOCTYPE , "ch") OR EXCLUDE (DOCTYPE , "ed") OR EXCLUDE (DOCTYPE , "sh") OR EXCLUDE (DOCTYPE , "no")) AND (LIMIT-TO (DOCTYPE , "ar"))

Cochrane Library

microRNA-21 or microRNA21 or miRNA-21 or miRNA21 or miR-21 or miR21 or microRNA or miRNA or miR in All Text AND prostate or prostatic in Title Abstract Keyword AND cancer or carcinoma or tumour or tumor or neoplasm or adenocarcinoma or PRAD in Title Abstract Keyword AND biomarker or marker or prognostic or prognosis or survival in Title Abstract Keyword

ST 2: Data items included in Covidence data extraction form (Adapted from CHARMS-PF checklist [22])

**General information**
Study ID
Title
Lead author and contact details
Country in which the study conducted
Study funding sources
Possible conflicts of interest for study authors
Notes
**Source of data**
Source of data (e.g., cohort, case control, randomised trial or registry data)
**Participants**
Participant eligibility and recruitment method
Participant description
Details of treatments received (if relevant)
Study dates
**Outcomes to be predicted**
Definition and method for measurement of outcomes
Was the same outcome definition (and method for measurement) used in all participants?
Types of outcomes
Were the outcomes assessed without knowledge of the candidate prognostic factors (i.e., blinded)?
Were candidate prognostic factors part of the outcome?
Time of outcome occurrence or summary of duration of follow-up
**Prognostic factors (index and comparator)**
Number and type of prognostic factors
Definition and method for measurement of prognostic factors
Timing of prognostic factor measurement
Were prognostic factors assessed blinded for outcome, and for each other (if relevant)?
Handling of prognostic factors in the analysis
**Sample size**
Was a sample size calculation conducted and, if so, how?
Number of participants and number of outcomes or events
Number of outcomes or events in relation to the number of candidate prognostic factors (events per variable)
**Missing data**
Number of participants with any missing value
Number of participants with missing data for miR-21 expression
Details of attrition (loss to follow-up) and, for time-to-event outcomes, number of censored observations
Handling of missing data
**Analysis (N/A for studies excluded from meta-analysis)**
Modelling method
How modelling assumptions were checked; the method for assessing non-proportional hazards
Method for selection of prognostic factors for inclusion in multivariable modelling
Method for selection or exclusion of prognostic factors during multivariable modelling, and criteria used for any selection or exclusion
Method of handling each continuous prognostic factor, including values of any cut points used and their justification
**Results of studies included in meta-analysis**
Unadjusted and adjusted prognostic effect estimates for miR-21 expression, the corresponding 95% confidence interval with p-value.
For the extracted adjusted prognostic effect estimate of interest, the set of adjustment factors used
**Results of studies excluded from meta-analysis**
Prognostic factors or stratification used for association analysis
Type of association analysis and estimates with p-value
**Interpretation and discussion**
Interpretation of presented results
Comparison with other studies, discussion of generalisability, strengths and limitations

ST 3: Records of authors contacted (12 studies)

Study ID	Author contacted	Response	Additional data
**Bryant2012** [36]	Freddie Hamdy <freddie.hamdy@nds.ox.ac.uk> Richard Bryant <richard.bryant@nds.ox.ac.uk>	Yes	miR-21 raw data excel file including 78 PCa patients
**Fendler2011** [40]	Klaus Jung <klaus.jung@charite.de>	Yes	No (Communication stopped without useful data)
**Huang2015a** [46]	Liang Wang <liwang@mcw.edu>	No	
**Kelly2015** [52]	Brian Kelly <drbriankelly@hotmail.com>	Yes	No (Communication stopped without useful data)
**Leite2013** [59]	Katia Ramos Moreira Leite Updated: <katiaramos@usp.br>	Yes	Clarification on results reported
**Leite2015** [60]			Details of multivariate analysis
**Lin2014** [64]	Hui-Ming Lin <h.lin@garvan.org.au>	Yes	Clarification on analysis method
**Lin2017** [65]			Results of univariate & multivariate analyses
**McDonald2019** [67]	Alicia McDonald <amcdonald3@phs.psu.edu>	Yes	No (miR-21 measured but not analysed because it did not meet criteria)
**Mortensen2014** [69]	Lars Dyrskjøt Andersen <lars@clin.au.dk>	Yes	Raw unanalysed data
**Schubert2013** [76]	Maria Schubert <schubert_m@klinik.uni-wuerzburg.de> Burkhard Kneitz <kneitz_b@klinik.uni-wuerzburg.de>	No	
**Stuopelyte2016** [81]	Sonata Jarmalaite <sonata.jarmalaite@gf.vu.lt> <sonata.jarmalaite@nvi.lt>	No	

ST 4: QUIPS (Quality in Prognostic Factor Studies) risk of bias classification tool [26]

	QUIPS domains		
**Signalling items**	**1. Study participation**<br>(a) Adequate participation in the study by eligible persons<br>(b) Description of the target population or population of interest<br>(c) Description of the baseline study sample<br>(d) Adequate description of the sampling frame and recruitment<br>(e) Adequate description of the period and place of recruitment<br>(f) Adequate description of inclusion and exclusion criteria		
**Risk of bias ratings ***	HIGH	MODERATE	LOW
	The relationship between the PF and outcome is very likely to be different for participants and eligible non-participants	The relationship between the PF and outcome may be different for participants and eligible non-participants	The relationship between the PF and outcome is unlikely to be different for participants and eligible non-participants
**Signalling items**	**2. Study attrition**<br>(a) Adequate response rate for study participants<br>(b) Description of attempts to collect information on participants who dropped out<br>(c) Reasons for loss to follow-up are provided<br>(d) Adequate description of participants lost to follow-up<br>(e) There are no important differences between participants who completed the study and those who did not		
**Risk of bias ratings ***	HIGH	MODERATE	LOW
	The relationship between the PF and outcome is very likely to be different for completing and non-completing participants	The relationship between the PF and outcome may be different for completing and non-completing participants	The relationship between the PF and outcome is unlikely to be different for completing and non-completing participants
**Signalling items**	**3. Prognostic factor measurement**<br>(a) A clear definition or description of the PF is provided<br>(b) Method of PF measurement is adequately valid and reliable<br>(c) Continuous variables are reported or appropriate cut-points are used<br>(d) The method and setting of measurement of PF is the same for all study participants<br>(e) Adequate proportion of the study sample has complete data for the PF<br>(f) Appropriate methods of imputation are used for missing PF data		
**Risk of bias ratings ***	HIGH	MODERATE	LOW
	The measurement of the PF is very likely to be different for different levels of the outcome of interest	The measurement of the PF may be different for different levels of the outcome of interest	The measurement of the PF is unlikely to be different for different levels of the outcome of interest
**Signalling items**	**4. Outcome measurement**<br>(a) A clear definition of the outcome is provided<br>(b) Method of outcome measurement used is adequately valid and reliable<br>(c) The method and setting of outcome measurement is the same for all study participants		
**Risk of bias ratings ***	HIGH	MODERATE	LOW
	The measurement of the outcome is very likely to be different related to the baseline level of the PF	The measurement of the outcome may be different related to the baseline level of the PF	The measurement of the outcome is unlikely to be different related to the baseline level of the PF
**Signalling items**	**5. Adjustment for covariates**<br>(a) All other important covariates are measured<br>(b) Clear definitions of the important covariates measured are provided<br>(c) Measurement of all important covariates is adequately valid and reliable<br>(d) The method and setting of covariate measurement are the same for all study participants<br>(e) Appropriate methods are used to deal with missing values of covariates, such as multiple imputation<br>(f) Important covariates are accounted for in the study design<br>(g) Important covariates are accounted for in the analysis		
**Risk of bias ratings ***	HIGH	MODERATE	LOW
	The observed effect of the covariate on the outcome is very likely to be distorted by another factor related to PF and outcome	The observed effect of the covariate on outcome may be distorted by another factor related to PF and outcome	The observed effect of the covariate on outcome is unlikely to be distorted by another factor related to PF and outcome
**Signalling items**	**6. Statistical analysis and reporting**<br>(a) Sufficient presentation of data to assess the adequacy of the analytic strategy<br>(b) Strategy for model building is appropriate and is based on a conceptual framework or model<br>(c) The selected statistical model is adequate for the design of the study<br>(d) There is no selective reporting of results		
**Risk of bias ratings ***	HIGH	MODERATE	LOW
	The reported results are very likely to be spurious or biased related to analysis or reporting	The reported results may be spurious or biased related to analysis or reporting	The reported results are unlikely to be spurious or biased related to analysis or reporting

***** Risk of bias is rated as **Unclear** when there is insufficient information to inform judgment.

PF: Prognostic factor

ST 5: Reasons for exclusion of 13 full-text articles

Reason for exclusion	Full-text articles
No prognostic data (n=8)	Benoist2020; Egidi2013; Li2015; Liu2018; Martens-Uzunova2012; Osipov2016; Valera2020; Yang2015
miR-21 not studied (n=4)	Haldrup2014; Knyazev2016; Moltzahn2011; Nam2015
Non-original human prognostic data (n=1)	Kumar2018

Benoist2020
Benoist, G.E., van Oort, I.M., Boerrigter, E., Verhaegh, G.W., van Hooij, O., Groen, L., Smit, F., de Mol, P., Hamberg, P., Dezentjé, V.O. and Mehra, N., 2020. Prognostic Value of Novel Liquid Biomarkers in Patients with Metastatic Castration-Resistant Prostate Cancer Treated with Enzalutamide: A Prospective Observational Study. *Clinical Chemistry*, *66*(6), pp.842-851.

Egidi2013
Egidi, M.G., Cochetti, G., Serva, M.R., Guelfi, G., Zampini, D., Mechelli, L. and Mearini, E., 2013. Circulating microRNAs and kallikreins before and after radical prostatectomy: are they really prostate cancer markers?. *BioMed research international*, *2013*.

Haldrup2014
Haldrup, C., Kosaka, N., Ochiya, T., Borre, M., Høyer, S., Orntoft, T.F. and Sorensen, K.D., 2014. Profiling of circulating microRNAs for prostate cancer biomarker discovery. *Drug delivery and translational research*, *4*(1), pp.19-30.

Knyazev2016
Knyazev, E., Samatov, T., Fomicheva, K., Nyushko, K., Alekseev, B. and Shkurnikov, M., 2016. MicroRNA hsa-miR-4674 in hemolysis-free blood plasma is associated with distant metastases of prostatic cancer. *Bulletin of Experimental Biology & Medicine*, *161*(1).

Kumar2018
Kumar, B., Rosenberg, A.Z., Choi, S.M., Fox-Talbot, K., De Marzo, A.M., Nonn, L., Brennen, W.N., Marchionni, L., Halushka, M.K. and Lupold, S.E., 2018. Cell-type specific expression of oncogenic and tumor suppressive microRNAs in the human prostate and prostate cancer. *Scientific reports*, *8*(1), pp.1-13.

Li2015
Li, M., Rai, A.J., DeCastro, G.J., Zeringer, E., Barta, T., Magdaleno, S., Setterquist, R. and Vlassov, A.V., 2015. An optimized procedure for exosome isolation and analysis using serum samples: application to cancer biomarker discovery. *Methods*, *87*, pp.26-30.

Liu2018
Liu, R.S., Olkhov-Mitsel, E., Jeyapala, R., Zhao, F., Commisso, K., Klotz, L., Loblaw, A., Liu, S.K., Vesprini, D., Fleshner, N.E. and Bapat, B., 2018. Assessment of serum microRNA biomarkers to predict reclassification of prostate cancer in patients on active surveillance. *The Journal of urology*, *199*(6), pp.1475-1481.

Martens-Uzunova2012
Martens-Uzunova, E.S., Jalava, S.E., Dits, N.F., Van Leenders, G.J.L.H., Møller, S., Trapman, J., Bangma, C.H., Litman, T., Visakorpi, T. and Jenster, G., 2012. Diagnostic and prognostic signatures from the small non-coding RNA transcriptome in prostate cancer. *Oncogene*, *31*(8), pp.978-991.

Moltzahn2011
Moltzahn, F., Olshen, A.B., Baehner, L., Peek, A., Fong, L., Stöppler, H., Simko, J., Hilton, J.F., Carroll, P. and Blelloch, R., 2011. Microfluidic-based multiplex qRT-PCR identifies diagnostic and prognostic microRNA signatures in the sera of prostate cancer patients. *Cancer research*, *71*(2), pp.550-560.

Nam2015
Nam, R.K., Amemiya, Y., Benatar, T., Wallis, C.J., Stojcic-Bendavid, J., Bacopulos, S., Sherman, C., Sugar, L., Naeim, M., Yang, W. and Zhang, A., 2015. Identification and validation of a five microRNA signature predictive of prostate cancer recurrence and metastasis: a cohort study. *Journal of Cancer*, *6*(11), p.1160.

Osipov2016
Osipov, I.D., Zaporozhchenko, I.A., Bondar, A.A., Zaripov, M.M., Voytsitskiy, V.E., Vlassov, V.V., Laktionov, P.P. and Morozkin, E.S., 2016. Cell-free miRNA-141 and miRNA-205 as prostate cancer biomarkers. In *Circulating Nucleic Acids in Serum and Plasma–CNAPS IX* (pp. 9-12). Springer, Cham.

Valera2020
Valera, V.A., Parra-Medina, R., Walter, B.A., Pinto, P. and Merino, M.J., 2020. microRNA expression profiling in young prostate cancer patients. *Journal of Cancer*, *11*(14), p.4106.

Yang2015
Yang, C.H., Pfeffer, S.R., Sims, M., Yue, J., Wang, Y., Linga, V.G., Paulus, E., Davidoff, A.M. and Pfeffer, L.M., 2015. The oncogenic microRNA-21 inhibits the tumor suppressive activity of FBXO11 to promote tumorigenesis. *Journal of Biological Chemistry*, *290*(10), pp.6037-6046.

ST 6: Characteristics of included studies and references (n=64)

Ref no.	Study ID	Study size	miR-21 source	miR-21 -5p/-3p	Comparator	Association
29	Agaoglu2011	51	plasma	Not specified	PSA, metastasis	Correlation, median diff
30	Al-Qatati2017	79	plasma	miR-21-5p	GS, pT, PSA, risk groups	FC
**31**	**Amankwah 2013**	65	tissue	Not specified	Aggressiveness (determined by GS or stage), recurrence (BCR/clinical metastasis/PCa death)	% diff
32	Arisan2020	40	tissue	Not specified	GS	% diff
33	Bell2015 *	43	tissue	Not specified	*(Raw data of m-R-21 in GEO not analysed. No other miR-21 data available.)*	
34	Bonci2016	15	tissue	Not specified	Metastasis	% diff
35	Brase2011	21	serum	Not specified	Metastasis	FC
36	Bryant2012 *	78	plasma	Not specified	*(Author provided miR-21 raw data excel file.)*	
37	Danarto2020	60	urine exosome	miR-21-5p	Metastasis	Mean diff
38	Endzeliņš 2017 *	50	plasma or exosome	miR-21-5p	*(Comparison and ROC curve of miR-21 expression between GS≥8 & ≤6 were done but not shown due to insignificant result.)*	
39	Farran2018	114	plasma	Not specified	Aggressiveness (determined by GS)	OR
40	Fendler2011 *	52	tissue	Not specified	*(Communication with authors failed to obtain full list of differentially expressed miRNAs.)*	
41	Foj2017	60	urine, urine exosome	miR-21-5p	GS, D'Amico risk groups	Mean diff
**42**	**Guan2016**	85	tissue	Not specified	GS, PSA, metastasis, age	Correlation
43	Gurbuz2020	65	whole blood	Not specified	GS, TNM, PSA	FC diff
44	Hart2014	20	tissue	Not specified	pT	FC diff
45	Hoey2019	75	serum	miR-21-5p	Risk groups	FC
46	Huang2015a *	Screening =23 Follow-up =100	plasma exosome	miR-21-5p	*(miR-21 raw data in supplemental materials; overall survival might have been analysed but contact author failed.)*	
47	Huang2015b	75	PBMC	Not specified	pT, cT, pN, metastasis, recurrence, age	Mean diff
48	Ibrahim2019a	100	plasma	Not specified	GS, pT, metastasis, DRE, prostate volume	Correlation, mean diff
49	Ibrahim2019b	80	plasma	Not specified	GS, pT, PSA, metastasis, DRE, prostate volume	Median diff
50	Ju2019	88	serum	Not specified	GS, pT, PSA, metastasis, BCR, risk groups	Mean diff
51	Katz2014	51	tissue	Not specified	GS, pT, PSA, BCR, risk groups	Mean diff
52	Kelly2015 *	75	whole blood	Not specified	*(miR-21 was among the 12 selected for expression profiling, but data wasn't presented. Author stopped communication.)*	
53	Kopcalic2019	15	PBMC	Not specified	Acute genitourinary radiotoxicity	Mean diff
54	Kotb2014	10	serum	Not specified	GS	Correlation
55	Kristensen 2016	Training =134 Validation	tissue	miR-21-3p	GS, BCR	FC, correlation

		=138				
56	Kurul2019	45	tissue	Not specified	Gleason upgrade, BCR	FC diff
57	Leite2011a	22	tissue	Not specified	Metastasis	Mean diff
58	Leite2011b	49	tissue	Not specified	BCR	Mean diff
59	Leite2013 **	48	tissue	Not specified	Risk groups (favourable vs non-favourable)	Mean diff
**60**	**Leite2015**	Discovery =53 Validation =127	tissue	miR-21-5p, miR-21-3p	BCR	FC, mean diff
**61**	**Li2012**	168	tissue	Not specified	GS, pT, PSA, pN, BCR, age, surgical margin, capsular invasion, organ confined disease	% diff
62	Lichner2013	Discovery =41 Validation =64	tissue	miR-21-5p, miR-21-3p	Risk groups	FC
63	Lichner2015	Discovery =45 Validation =60	tissue	miR-21-5p, miR-21-3p	GG	FC
**64**	**Lin2014 ***	97	plasma or serum	Not specified	*(Pre-docetaxel median diff and post-docetaxel median FC in responder vs non-responder compared. Results for miR-21 not shown due to insignificant p-values.)*	
**65**	**Lin2017 ***	87	plasma	Not specified	*(No association analysis with comparator.)*	
66	Long2011 *	Training =70 Validation =40	tissue	Not specified	*(miR-21 expression relating to BCR prediction raw data in supplemental materials.)*	
67	McDonald 2019 *	66	plasma	Not specified	*(miR-21 expression measured but not analysed because it did not meet study criteria.)*	
**68**	**Melbø-Jørgensen 2014**	535	tissue	miR-21-5p	GS, pT, BCR, perineural infiltration, vascular infiltration	Correlation, FC
69	Mortensen2014 *	36	tissue	Not specified	*(miR-21 expression measured but not analysed.)*	
70	Nam2018 *	38	tissue	miR-21-5p, miR-21-3p	*(miR-21 normalised read count available in GEO, not analysed.)*	
71	Ostano2020	48	tissue	miR-21-3p	Neuroendocrine-like vs Adeno PCa	FC
72	Reis2012	53	tissue	Not specified	GS, pT, PSA, BCR	Mean diff
73	Ren2014	204	tissue	Not specified	GS, pT, metastasis, BCR, age, ethnicity, survival, tissue type, hormone therapy	FC, mean diff
74	Samaan2014	95	Not stated	Not specified	GG	FC
75	Sapre2014	36	urine	Not specified	Risk groups	Ct FC
76	Schubert 2013 *	13	tissue	Not specified	*(miR-21 tested in microarray; raw data deposited in GEO (GSE18671); not included in further tests because of insignificant differential expression in high-risk PCa compared to BPH.)*	
77	Selth2013	Screening	serum	Not specified	BCR	FC

		=16 Validation =70				
**78**	**Sharova2021**	31	plasma	miR-21-5p	Haemoglobin; Neutrophil/lymphocyte ratio; PSA; Time to CRPC	Correlation
79	Shen2012	82	plasma	Not specified	GS, pT, PSA, BCR, risk groups (CAPRA, D'Amico), age, prostate volume, ethnicity, follow-up time, family history of PCa	Mean diff (copy number)
80	Singh2014	93	serum	Not specified	Biochemical progression	Mean diff (delta Ct)
81	Stuopelyte 2016	143	urine	Not specified	GS, pT, BCR	FC
82	Suer2019	40	tissue	miR-21-3p	BCR	FC
83	Watahiki2013	50	plasma	Not specified	mCRPC	Mean diff
**84**	**Yang2016**	92	PBMC	Not specified	GS, cT, PSA, metastasis (bone), BCR, age	Mean diff
**85**	**Zedan2017**	49	tissue	Not specified	GS, pT, PSA, risk groups (D'Amico, NCCN)	Correlation
86	Zedan2018	Screening =46 Validation =149	tissue or plasma	Not specified	GS, PSA	Mean diff
87	Zedan2019	149	plasma	Not specified	GS, cT, PSA, risk groups (EAU), age, prostate volume	Correlation
88	Zhang2011	50	serum	Not specified	Chemo-resistance	
**89**	**Zhao2019a**	206	tissue	miR-21-5p	ISUP (based on GS), pT, PSA, age, DRE, margin	Correlation
90	Zhao2019b	103	urine	Not specified	PSA, age, %core, reclassification	Correlation
91	Zheng2014	118	tissue	Not specified	Recurrence (BCR/local recurrence/systemic metastases/PCa death)	Mean diff, OR
92	Zhu2019	158	tissue	Not specified	Risk groups (identified by GAS5 SNPs)	FC

Studies in **bold** are eligible for meta-analyses (n=11).
Possible part overlap of participants between Ibrahim2019a [48] and Ibrahim2019b [49].
* miR-21 expression measured but no useful data for narrative summary (n=13).
** (Leite2013 [59]) A corrigendum would be published in *Urologic Oncology*.

ARTA: Androgen receptor-targeted agents; **BCR:** Biochemical recurrence; **BPH:** Benign prostate enlargement; **CAPRA:** Cancer of the Prostate Risk Assessment; **CRPC:** Castration-resistant prostate cancer; **cT:** Clinical tumour stage; **Ct:** Threshold cycle; **diff:** Difference; **DRE:** Digital rectal examination; **EAU:** European Association of Urology; **FC:** Fold change; **GAS5:** Growth Arrest Specific 5; **GEO:** Gene Expression Omnibus; **GG:** Gleason grade; **GS:** Gleason score; **ISUP:** International Society of Urological Pathology; **mCRPC:** Metastatic castration resistant prostate cancer; **miRNAs:** microRNAs; **NCCN:** National Comprehensive Cancer Network; **OR:** Odds ratio; **PBMC:** Peripheral blood mononuclear cell; **PCa:** Prostate cancer; **pN:** Lymph node metastasis; **PSA:** Prostate-specific antigen; **pT:** Pathological tumour stage; **ROC:** Receiver operating characteristic; **SNPs:** Single-nucleotide polymorphisms; **TNM:** Tumour, Node, Metastasis staging

Agaoglu2011
Agaoglu, F.Y., Kovancilar, M., Dizdar, Y., Darendeliler, E., Holdenrieder, S., Dalay, N. and Gezer, U., 2011. Investigation of miR-21, miR-141, and miR-221 in blood circulation of patients with prostate cancer. *Tumor Biology*, *32*(3), pp.583-588.

Al-Qatati2017
Al-Qatati, A., Akrong, C., Stevic, I., Pantel, K., Awe, J., Saranchuk, J., Drachenberg, D., Mai, S. and Schwarzenbach, H., 2017. Plasma microRNA signature is associated with risk stratification in prostate cancer patients. *International journal of cancer*, *141*(6), pp.1231-1239.

Amankwah2013 *(Analysis 1)*
Amankwah, E.K., Anegbe, E., Park, H., Pow-Sang, J., Hakam, A. and Park, J.Y., 2013. miR-21, miR-221 and miR-222 expression and prostate cancer recurrence among obese and non-obese cases. *Asian journal of andrology*, *15*(2), p.226.

Arisan2020
Arisan, E.D., Rencuzogullari, O., Freitas, I.L., Radzali, S., Keskin, B., Kothari, A., Warford, A. and Uysal-Onganer, P., 2020. Upregulated Wnt-11 and miR-21 expression trigger epithelial mesenchymal transition in aggressive prostate cancer cells. *Biology*, *9*(3), p.52.

Bell2015
Bell, E.H., Kirste, S., Fleming, J.L., Stegmaier, P., Drendel, V., Mo, X., Ling, S., Fabian, D., Manring, I., Jilg, C.A. and Schultze-Seemann, W., 2015. A novel miRNA-based predictive model for biochemical failure following post-prostatectomy salvage radiation therapy. *PloS one*, *10*(3), p.e0118745.

Bonci2016
Bonci, D., Coppola, V., Patrizii, M., Addario, A., Cannistraci, A., Francescangeli, F., Pecci, R., Muto, G., Collura, D., Bedini, R. and Zeuner, A., 2016. A microRNA code for prostate cancer metastasis. *Oncogene*, *35*(9), pp.1180-1192.

Brase2011
Brase, J.C., Johannes, M., Schlomm, T., Fälth, M., Haese, A., Steuber, T., Beissbarth, T., Kuner, R. and Sültmann, H., 2011. Circulating miRNAs are correlated with tumor progression in prostate cancer. *International journal of cancer*, *128*(3), pp.608-616.

Bryant2012
Bryant, R., Pawlowski, T., Catto, J.W.F., Marsden, G., Vessella, R.L., Rhees, B., Kuslich, C., Visakorpi, T. and Hamdy, F.C., 2012. Changes in circulating microRNA levels associated with prostate cancer. *British journal of cancer*, *106*(4), pp.768-774.

Danarto2020
Danarto, R., Astuti, I., Umbas, R. and Haryana, S.M., 2020. Urine miR-21-5p and miR-200c-3p as potential non-invasive biomarkers in patients with prostate cancer. *Turkish journal of urology*, *46*(1), p.26.

Endzeliņš2017
Endzeliņš, E., Berger, A., Melne, V., Bajo-Santos, C., Soboļevska, K., Ābols, A., Rodriguez, M., Šantare, D., Rudņickiha, A., Lietuvietis, V. and Llorente, A., 2017. Detection of circulating miRNAs: comparative analysis of extracellular vesicle-incorporated miRNAs and cell-free miRNAs in whole plasma of prostate cancer patients. *BMC cancer*, *17*(1), pp.1-13.

Farran2018
Farran, B., Dyson, G., Craig, D., Dombkowski, A., Beebe-Dimmer, J.L., Powell, I.J., Podgorski, I., Heilbrun, L., Bolton, S. and Bock, C.H., 2018. A study of circulating microRNAs identifies a new potential biomarker panel to distinguish aggressive prostate cancer. *Carcinogenesis*, *39*(4), pp.556-561.

Fendler2011
Fendler, A., Jung, M., Stephan, C., Honey, R.J., Stewart, R.J., Pace, K.T., Erbersdobler, A., Samaan, S., Jung, K. and Yousef, G.M., 2011. miRNAs can predict prostate cancer biochemical relapse and are involved in tumor progression. *International journal of oncology*, *39*(5), pp.1183-1192.

Foj2017
Foj, L., Ferrer, F., Serra, M., Arévalo, A., Gavagnach, M., Giménez, N. and Filella, X., 2017. Exosomal and non-exosomal urinary miRNAs in prostate cancer detection and prognosis. *The Prostate*, *77*(6), pp.573-583.

Guan2016 *(Eligible for meta-analysis but no similar studies)*
Guan, Y., Wu, Y., Liu, Y., Ni, J. and Nong, S., 2016. Association of microRNA-21 expression with clinicopathological characteristics and the risk of progression in advanced prostate cancer patients receiving androgen deprivation therapy. *The Prostate*, *76*(11), pp.986-993.

Gurbuz2020
Gurbuz, V., Kiliccioglu, I., Dikmen, A.U., Bilen, C.Y., Sozen, S. and Konac, E., 2020. Comparative analysis of epi-miRNA expression levels in local/locally advanced and metastatic prostate cancer patients. *Gene*, *758*, p.144963.

Hart2014
Hart, M., Nolte, E., Wach, S., Szczyrba, J., Taubert, H., Rau, T.T., Hartmann, A., Grässer, F.A. and Wullich, B., 2014. Comparative

microRNA profiling of prostate carcinomas with increasing tumor stage by deep sequencing. *Molecular cancer research*, *12*(2), pp.250-263.

Hoey2019
Hoey, C., Ahmed, M., Ghiam, A.F., Vesprini, D., Huang, X., Commisso, K., Commisso, A., Ray, J., Fokas, E., Loblaw, D.A. and He, H.H., 2019. Circulating miRNAs as non-invasive biomarkers to predict aggressive prostate cancer after radical prostatectomy. *Journal of translational medicine*, *17*(1), pp.1-11.

Huang2015a
Huang, X., Yuan, T., Liang, M., Du, M., Xia, S., Dittmar, R., Wang, D., See, W., Costello, B.A., Quevedo, F. and Tan, W., 2015. Exosomal miR-1290 and miR-375 as prognostic markers in castration-resistant prostate cancer. *European urology*, *67*(1), pp.33-41.

Huang2015b
Huang, W., Kang, X.L., Cen, S., Wang, Y. and Chen, X., 2015. High-level expression of microRNA-21 in peripheral blood mononuclear cells is a diagnostic and prognostic marker in prostate cancer. *Genetic testing and molecular biomarkers*, *19*(9), pp.469-475.

Ibrahim2019a
Ibrahim, N.H., Abdellateif, M.S., Thabet, G., Kassem, S.H., El-Salam, M.A., El-Leithy, A.A. and Selim, M.M., 2019. Combining PHI and miRNAs as biomarkers in prostate cancer diagnosis and prognosis. *Clin Lab*, *65*(7), p.10.

Ibrahim2019b *(Possible part overlap of participants with Ibrahim 2019a)*
Ibrahim, N.H., Abdellateif, M.S., Kassem, S.H.A., Abd El Salam, M.A. and El Gammal, M.M., 2019. Diagnostic significance of miR-21, miR-141, miR-18a and miR-221 as novel biomarkers in prostate cancer among Egyptian patients. *Andrologia*, *51*(10), p.e13384.

Ju2019
Ju, G., Lian, J., Wang, Z., Cao, W., Lin, J., Li, Y. and Yin, L., 2019. Correlation between miRNA-21 expression and diagnosis, metastasis and prognosis of prostate cancer. *International Journal of Clinical and Experimental Medicine*, *12*(7), pp.8172-8180.

Katz2014
Katz, B., Reis, S.T., Viana, N.I., Morais, D.R., Moura, C.M., Dip, N., Silva, I.A., Iscaife, A., Srougi, M. and Leite, K.R., 2014. Comprehensive study of gene and microRNA expression related to epithelial-mesenchymal transition in prostate cancer. *PloS one*, *9*(11), p.e113700.

Kelly2015
Kelly, B.D., Miller, N., Sweeney, K.J., Durkan, G.C., Rogers, E., Walsh, K. and Kerin, M.J., 2015. A circulating microRNA signature as a biomarker for prostate cancer in a high risk group. *Journal of clinical medicine*, *4*(7), pp.1369-1379.

Kopcalic2019
Kopcalic, K., Petrovic, N., Stanojkovic, T.P., Stankovic, V., Bukumiric, Z., Roganovic, J., Malisic, E. and Nikitovic, M., 2019. Association between miR-21/146a/155 level changes and acute genitourinary radiotoxicity in prostate cancer patients: A pilot study. *Pathology-Research and Practice*, *215*(4), pp.626-631.

Kotb2014
Kotb, S., Mosharafa, A., Essawi, M., Hassan, H., Meshref, A. and Morsy, A., 2014. Circulating miRNAs 21 and 221 as biomarkers for early diagnosis of prostate cancer. *Tumor Biology*, *35*(12), pp.12613-12617.

Kristensen2016
Kristensen, H., Thomsen, A.R., Haldrup, C., Dyrskjøt, L., Høyer, S., Borre, M., Mouritzen, P., Ørntoft, T.F. and Sørensen, K.D., 2016. Novel diagnostic and prognostic classifiers for prostate cancer identified by genome-wide microRNA profiling. *Oncotarget*, *7*(21), p.30760.

Kurul2019
Kurul, N.O., Ates, F., Yilmaz, I., Narli, G., Yesildal, C. and Senkul, T., 2019. The association of let-7c, miR-21, miR-145, miR-182, and miR-221 with clinicopathologic parameters of prostate cancer in patients diagnosed with low-risk disease. *The Prostate*, *79*(10), pp.1125-1132.

Leite2011a
Leite, K.R., Sousa-Canavez, J.M., Reis, S.T., Tomiyama, A.H., Camara-Lopes, L.H., Sañudo, A., Antunes, A.A. and Srougi, M., 2011, May. Change in expression of miR-let7c, miR-100, and miR-218 from high grade localized prostate cancer to metastasis. In *Urologic Oncology: Seminars and Original Investigations* (Vol. 29, No. 3, pp. 265-269). Elsevier.

Leite2011b
Leite, K.R., Tomiyama, A., Reis, S.T., Sousa-Canavez, J.M., Sañudo, A., Dall'Oglio, M.F., Camara-Lopes, L.H. and Srougi, M., 2011. MicroRNA-100 expression is independently related to biochemical recurrence of prostate cancer. *The Journal of urology*, *185*(3), pp.1118-1122.

Leite2013 *(A corrigendum will be published in Urologic Oncology)*

Leite, K.R., Tomiyama, A., Reis, S.T., Sousa-Canavez, J.M., Sañudo, A., Camara-Lopes, L.H. and Srougi, M., 2013, August. MicroRNA expression profiles in the progression of prostate cancer—from high-grade prostate intraepithelial neoplasia to metastasis. In *Urologic Oncology: Seminars and Original Investigations* (Vol. 31, No. 6, pp. 796-801). Elsevier.

Leite2015 *(Analysis 1)*
Leite, K.R., Reis, S.T., Viana, N., Morais, D.R., Moura, C.M., Silva, I.A., Pontes Jr, J., Katz, B. and Srougi, M., 2015. Controlling RECK miR21 promotes tumor cell invasion and is related to biochemical recurrence in prostate cancer. *Journal of Cancer*, *6*(3), p.292.

Li2012 *(Analysis 1)*
Li, T., Li, R.S., Li, Y.H., Zhong, S., Chen, Y.Y., Zhang, C.M., Hu, M.M. and Shen, Z.J., 2012. miR-21 as an independent biochemical recurrence predictor and potential therapeutic target for prostate cancer. *The Journal of urology*, *187*(4), pp.1466-1472.

Lichner2013
Lichner, Z., Fendler, A., Saleh, C., Nasser, A.N., Boles, D., Al-Haddad, S., Kupchak, P., Dharsee, M., Nuin, P.S., Evans, K.R. and Jung, K., 2013. MicroRNA signature helps distinguish early from late biochemical failure in prostate cancer. *Clinical chemistry*, *59*(11), pp.1595-1603.

Lichner2015
Lichner, Z., Ding, Q., Samaan, S., Saleh, C., Nasser, A., Al-Haddad, S., Samuel, J.N., Fleshner, N.E., Stephan, C., Jung, K. and Yousef, G.M., 2015. miRNAs dysregulated in association with Gleason grade regulate extracellular matrix, cytoskeleton and androgen receptor pathways. *The Journal of pathology*, *237*(2), pp.226-237.

Lin2014 *(Analysis 3)*
Lin, H.M., Castillo, L., Mahon, K.L., Chiam, K., Lee, B.Y., Nguyen, Q., Boyer, M.J., Stockler, M.R., Pavlakis, N., Marx, G. and Mallesara, G., 2014. Circulating microRNAs are associated with docetaxel chemotherapy outcome in castration-resistant prostate cancer. *British journal of cancer*, *110*(10), pp.2462-2471.

Lin2017 *(Analysis 3)*
Lin, H.M., Mahon, K.L., Spielman, C., Gurney, H., Mallesara, G., Stockler, M.R., Bastick, P., Briscoe, K., Marx, G., Swarbrick, A. and Horvath, L.G., 2017. Phase 2 study of circulating microRNA biomarkers in castration-resistant prostate cancer. *British journal of cancer*, *116*(8), pp.1002-1011.

Long2011
Long, Q., Johnson, B.A., Osunkoya, A.O., Lai, Y.H., Zhou, W., Abramovitz, M., Xia, M., Bouzyk, M.B., Nam, R.K., Sugar, L. and Stanimirovic, A., 2011. Protein-coding and microRNA biomarkers of recurrence of prostate cancer following radical prostatectomy. *The American journal of pathology*, *179*(1), pp.46-54.

McDonald2019
McDonald, A.C., Vira, M., Walter, V., Shen, J., Raman, J.D., Sanda, M.G., Patil, D. and Taioli, E., 2019. Circulating microRNAs in plasma among men with low-grade and high-grade prostate cancer at prostate biopsy. *The Prostate*, *79*(9), pp.961-968.

Melbø-Jørgensen2014 *(Analysis 1)*
Melbø-Jørgensen, C., Ness, N., Andersen, S., Valkov, A., Dønnem, T., Al-Saad, S., Kiselev, Y., Berg, T., Nordby, Y., Bremnes, R.M. and Busund, L.T., 2014. Stromal expression of MiR-21 predicts biochemical failure in prostate cancer patients with Gleason score 6. *PloS one*, *9*(11), p.e113039.

Mortensen2014
Mortensen, M.M., Høyer, S., Ørntoft, T.F., Sørensen, K.D., Dyrskjøt, L. and Borre, M., 2014. High miR-449b expression in prostate cancer is associated with biochemical recurrence after radical prostatectomy. *BMC cancer*, *14*(1), pp.1-7.

Nam2018
Nam, R.K., Wallis, C.J., Amemiya, Y., Benatar, T. and Seth, A., 2018. Identification of a novel MicroRNA panel associated with metastasis following radical prostatectomy for prostate cancer. *Anticancer research*, *38*(9), pp.5027-5034.

Ostano2020
Ostano, P., Mello-Grand, M., Sesia, D., Gregnanin, I., Peraldo-Neia, C., Guana, F., Jachetti, E., Farsetti, A. and Chiorino, G., 2020. Gene Expression Signature Predictive of Neuroendocrine Transformation in Prostate Adenocarcinoma. *International journal of molecular sciences*, *21*(3), p.1078.

Reis2012
Reis, S.T., Pontes-Junior, J., Antunes, A.A., Dall'Oglio, M.F., Dip, N., Passerotti, C.C., Rossini, G.A., Morais, D.R., Nesrallah, A.J., Piantino, C. and Srougi, M., 2012. miR-21 may acts as an oncomir by targeting RECK, a matrix metalloproteinase regulator, in prostate cancer. *BMC urology*, *12*(1), pp.1-7.

Ren2014
Ren, Q., Liang, J., Wei, J., Basturk, O., Wang, J., Daniels, G., Gellert, L.L., Li, Y., Shen, Y., Osman, I. and Zhao, J., 2014. Epithelial and stromal expression of miRNAs during prostate cancer progression. *American journal of translational research*, *6*(4), p.329.

Samaan2014

Samaan, S., Lichner, Z., Ding, Q., Saleh, C., Samuel, J., Streutker, C. and Yousef, G.M., 2014. Kallikreins are involved in an miRNA network that contributes to prostate cancer progression. *Biological chemistry*, *395*(9), pp.991-1001.

Sapre2014
Sapre, N., Hong, M.K., Macintyre, G., Lewis, H., Kowalczyk, A., Costello, A.J., Corcoran, N.M. and Hovens, C.M., 2014. Curated microRNAs in urine and blood fail to validate as predictive biomarkers for high-risk prostate cancer. *PloS one*, *9*(4), p.e91729.

Schubert2013
Schubert, M., Spahn, M., Kneitz, S., Scholz, C.J., Joniau, S., Stroebel, P., Riedmiller, H. and Kneitz, B., 2013. Distinct microRNA expression profile in prostate cancer patients with early clinical failure and the impact of let-7 as prognostic marker in high-risk prostate cancer. *PloS one*, *8*(6), p.e65064.

Selth2013
Selth, L.A., Townley, S.L., Bert, A.G., Stricker, P.D., Sutherland, P.D., Horvath, L.G., Goodall, G.J., Butler, L.M. and Tilley, W.D., 2013. Circulating microRNAs predict biochemical recurrence in prostate cancer patients. *British journal of cancer*, *109*(3), pp.641-650.

Sharova, 2021
Sharova, E., Maruzzo, M., Del Bianco, P., Cavallari, I., Pierantoni, F., Basso, U., Ciminale, V. and Zagonel, V., 2021 Prognostic Stratification of Metastatic Prostate Cancer Patients Treated With Abiraterone and Enzalutamide Through an Integrated Analysis of Circulating Free microRNAs and Clinical Parameters. *Frontiers in Oncology,* 11: 626104.

Shen2012
Shen, J., Hruby, G.W., McKiernan, J.M., Gurvich, I., Lipsky, M.J., Benson, M.C. and Santella, R.M., 2012. Dysregulation of circulating microRNAs and prediction of aggressive prostate cancer. *The Prostate*, *72*(13), pp.1469-1477.

Singh2014
Singh, P.K., Preus, L., Hu, Q., Yan, L., Long, M.D., Morrison, C.D., Nesline, M., Johnson, C.S., Koochekpour, S., Kohli, M. and Liu, S., 2014. Serum microRNA expression patterns that predict early treatment failure in prostate cancer patients. *Oncotarget*, *5*(3), p.824.

Stuopelytė2016
Stuopelytė, K., Daniūnaitė, K., Jankevičius, F. and Jarmalaitė, S., 2016. Detection of miRNAs in urine of prostate cancer patients. *Medicina*, *52*(2), pp.116-124.

Suer2019
Suer, I., Guzel, E., Karatas, O.F., Creighton, C.J., Ittmann, M. and Ozen, M., 2019. MicroRNAs as prognostic markers in prostate cancer. *The Prostate*, *79*(3), pp.265-271.

Watahiki2013
Watahiki, A., Macfarlane, R.J., Gleave, M.E., Crea, F., Wang, Y., Helgason, C.D. and Chi, K.N., 2013. Plasma miRNAs as biomarkers to identify patients with castration-resistant metastatic prostate cancer. *International journal of molecular sciences*, *14*(4), pp.7757-7770.

Yang2016 *(Analysis 3)*
Yang, B., Liu, Z., Ning, H., Zhang, K., Pan, D., Ding, K., Huang, W., Kang, X.L., Wang, Y. and Chen, X., 2016. MicroRNA-21 in peripheral blood mononuclear cells as a novel biomarker in the diagnosis and prognosis of prostate cancer. *Cancer biomarkers*, *17*(2), pp.223-230.

Zedan2017 *(Analysis 2)*
Zedan, A.H., Blavnsfeldt, S.G., Hansen, T.F., Nielsen, B.S., Marcussen, N., Pleckaitis, M., Osther, P.J.S. and Sørensen, F.B., 2017. Heterogeneity of miRNA expression in localized prostate cancer with clinicopathological correlations. *PLoS One*, *12*(6), p.e0179113.

Zedan2018
Zedan, A.H., Hansen, T.F., Assenholt, J., Pleckaitis, M., Madsen, J.S. and Osther, P.J.S., 2018. microRNA expression in tumour tissue and plasma in patients with newly diagnosed metastatic prostate cancer. *Tumor Biology*, *40*(5), p.1010428318775864.

Zedan2019
Zedan, A.H., Hansen, T.F., Assenholt, J., Madsen, J.S. and Osther, P.J., 2019. Circulating miRNAs in localized/locally advanced prostate cancer patients after radical prostatectomy and radiotherapy. *The Prostate*, *79*(4), pp.425-432.

Zhang2011
Zhang, H.L., Yang, L.F., Zhu, Y., Yao, X.D., Zhang, S.L., Dai, B., Zhu, Y.P., Shen, Y.J., Shi, G.H. and Ye, D.W., 2011. Serum miRNA-21: Elevated levels in patients with metastatic hormone-refractory prostate cancer and potential predictive factor for the efficacy of docetaxel-based chemotherapy. *The prostate*, *71*(3), pp.326-331.

Zhao2019a *(Analysis 2)*
Zhao, Z., Weickmann, S., Jung, M., Lein, M., Kilic, E., Stephan, C., Erbersdobler, A., Fendler, A. and Jung, K., 2019. A novel predictor tool of biochemical recurrence after radical prostatectomy based on a five-microRNA tissue

signature. *Cancers*, *11*(10), p.1603.

Zhao2019b

Zhao, F., Vesprini, D., Liu, R.S., Olkhov-Mitsel, E., Klotz, L.H., Loblaw, A., Liu, S.K. and Bapat, B., 2019, May. Combining urinary DNA methylation and cell-free microRNA biomarkers for improved monitoring of prostate cancer patients on active surveillance. In *Urologic Oncology: Seminars and Original Investigations* (Vol. 37, No. 5, pp. 297-e9). Elsevier.

Zheng2014

Zheng, Q., Peskoe, S.B., Ribas, J., Rafiqi, F., Kudrolli, T., Meeker, A.K., De Marzo, A.M., Platz, E.A. and Lupold, S.E., 2014. Investigation of miR-21, miR-141, and miR-221 expression levels in prostate adenocarcinoma for associated risk of recurrence after radical prostatectomy. *The Prostate*, *74*(16), pp.1655-1662.

Zhu2019

Zhu, L., Zhu, Q., Wen, H., Huang, X. and Zheng, G., 2019. Mutations in GAS5 affect the transformation from benign prostate proliferation to aggressive prostate cancer by affecting the transcription efficiency of GAS5. *Journal of cellular physiology*, *234*(6), pp.8928-8940.

ST 7: Rationales for rating down certainty of evidence - GRADE

Domains	Analysis 1.1	Analysis 1.2	Analysis 2	Analysis 3	Analysis 4.1	Analysis 4.2
**RoB**	Estimate was unadjusted but sensitivity analysis showed limited difference in HR, rate-down not necessary.<br><br>High RoB in 3 studies (Amankwah2013 – Domain 1, Leite2015 & Li2012 – Domain 6), rate down 1 point.	Visual inspection of the point estimates and CI showed limited difference caused by difference in covariate adjustments, rate-down not necessary.<br><br>High RoB in 3 studies (Amankwah2013 – Domain 1, Leite2015 & Li2012 – Domain 6), rate down 1 point.	Unadjusted estimate and high RoB in 1 study (Zhao2019a – Domain 5), rate down 1 point.	Unadjusted estimate and high RoB in 3 studies (Lin2014, Lin2017 & Sharova2021 – Domain 5), rate down 1 point.	High RoB in both studies (Domain 5), rate down 1 point.	High RoB in both studies (Domain 5), rate down 1 point.
**Inconsistency**	Amankwah2013 outlying but low weight (8.5%), rate-down not necessary.	Amankwah2013 outlying but low weight (8.5%), rate-down not necessary.	Both studies showed positive association and CI overlapped, no rate-down.	Sharova2021 outlying but low weight (8.2%), rate-down not necessary.	The two studies showed opposite direction results, rate down 1 point.	The two studies showed opposite direction results, rate down 1 point.
**Indirectness**	Amankwah2013 RFS endpoint included clinical metastasis and PCa death but low weight, rate-down not necessary.	Amankwah2013 RFS endpoint included clinical metastasis and PCa death but low weight, rate-down not necessary.	No rate-down.	Lin2014 & Lin2017 included CRPC patients, not representing entire PCa population; main aim was to address chemo-response, rate down 1 point.	No rate-down.	No rate-down.
**Imprecision**	Pooled CI well excluded HR of 1 but individual HRs were not reported and hence estimated from available data, rate down 1 point.	Pooled CI well excluded HR of 1, no rate-down.	Pooled CI close to HR of 1 (CI: 1.01-1.26), rate down 1 point.	HR was not reported and hence estimated from available data in Yang 2016. Pooled CI close to HR of 1 (CI: 1.06-2.01), rate down 1 point.	Wide pooled CI crossing HR of 1 (CI: 0.63-1.88), rate down 1 point.	Wide pooled CI crossing HR of 1 (CI: 0.70-2.27), rate down 1 point.
**Publication bias**	Publication bias was not assessed because there was inadequate number of studies for proper assessment by funnel plot and statistical tests.					
**Overall certainty**	LOW	MODERATE	LOW	VERY LOW	VERY LOW	VERY LOW

CI: Confidence interval; **CRPC:** Castration-resistant prostate cancer; **HR:** Hazard ratio; **mCRPC:** metastatic castration-resistant prostate cancer; **PCa:** Prostate cancer; **RFS:** Recurrence-free survival; **RoB:** Risk of bias
